# Alpha Neurofeedback Training in Elite Soccer Players Trained in Groups

**DOI:** 10.1007/s10484-024-09654-1

**Published:** 2024-08-10

**Authors:** Geert J. M. van Boxtel, Ad J. J. M. Denissen, Joep A. de Groot, Marjolein S. Neleman, Jur Vellema, Evelijne M. Hart de Ruijter

**Affiliations:** 1 Department of Cognitive Neuropsychology, Tilburg University, Warandelaan 2, P. O. Box 90153, 5000 LE Tilburg, 5037 AB, Tilburg, The Netherlands; 2 Philips Research, Eindhoven, The Netherlands; 3 Alphabeats, Eindhoven, The Netherlands; 4 Kliniek ViaSana, Mill, The Netherlands

**Keywords:** Alpha activity, Neurofeedback training, Soccer, Cognitive performance, Wearable EEG

## Abstract

Neurofeedback training is applied in the world of sports as a means to improve athletes’ performance. Training sessions are usually organized on an individual basis, one at a time. Here we investigated if the training could also be organized in groups. Forty-one national-level football (soccer) players (26 females, 15 males) carried out training sessions simultaneously in groups of up to 13, using a wearable device with Bluetooth connection, during their regular training hours at the club. It was possible to obtain good EEG measurements using this setup, albeit with a somewhat higher data loss than usual in standard laboratory sessions. The brain’s alpha activity was trained using music-based neurofeedback in a crossover design. A training session consisted of alternating periods of neurofeedback and execution of cognitive tasks. EEG alpha (8–12 Hz) activity was higher in the neurofeedback periods compared to the cognitive task periods, and the reverse was true for beta (13–30 Hz) activity. The training program resulted in an increase of 34% in alpha activity associated with the training, and improved the athletes’ performance on task switching and mental rotation tasks. In addition, self-reported sleep duration, as well as scores on the Being in Shape questionnaire (Feeling of Control and Flow) also improved. This study shows that neurofeedback training is feasible in groups of athletes, which can stimulate its application in team sports.

**Supplementary Information:**

The online version contains supplementary material available at 10.1007/s10484-024-09654-1.

## Introduction

Elite athletes try to control every single aspect of their performance to reach the highest level possible. The concept of “marginal gains” (e.g., Hall et al., 2012) concerns the idea that even small improvements on a particular detail such as attention, speed of action, processing speed, can mean the difference between a gold and silver medal. It is therefore no surprise that a considerable amount of research is devoted to optimizing sports performance. In addition to control over physical aspects of the sport, it is also important for top athletes to gain control over the mental aspects that play a role in optimal sports performance. Sports psychology is a rapidly expanding branch within sports science, and a number of positive (e.g., mindfulness, mental practice, neurofeedback) as well as negative (anxiety, anger, depression) influences on sports performance have been identified (Lochbaum et al., 2022).

In addition to behavioral assessment and intervention tools, psychophysiological measures are increasingly being used in the field of sports performance. These measurements are non-intrusive, easy to apply, have a good time resolution, and allow the estimation of both central and autonomic nervous system activity. Many autonomic measures cardiovascular activity can nowadays be measured quite easily using standard wearable devices. However, if the aim is to monitor or influence mental aspects of sports performance, it would be more obvious to look at central nervous system activity instead of autonomic nervous system activity as they are measured by standard wearable devices. Unsupervised measurement of central nervous system activity is much more difficult though, and therefore much less being used at the moment compared to autonomic measures.

The electroencephalogram (EEG), first discovered around 100 years ago, could for a long time only be recorded under laboratory conditions in an electrically shielded room from participants sitting as still as possible wearing head caps containing conductive gel. Movement, electrical interference, or bad electrode contacts would make the EEG very hard to interpret. This made EEG applications during sports performance virtually impossible, and applications around sports events cumbersome. Nevertheless, EEG technology has been used for some time in a variety of sports, mostly in individual sports such as golf and archery, and the general findings are that a decrease in brain activation is related to an increase in sports performance. This “neural efficiency hypothesis”, developed in the context of general intelligence (Haier et al., 1988), states that experts perform more effectively than beginners by only recruiting the brain areas needed to perform the task at hand, while at the same time inhibiting other brain areas that are not required.

Rhythmic EEG activity can be classified into several frequency bands: delta (< 4 Hz), theta (4–7 Hz), alpha (8–12 Hz), beta (13–30 Hz), and gamma (> 30 Hz). Fast EEG rhythms are indicative of brain activation, as in intense mental activity; slow rhythms with brain inactivation, as in sleep. The intermediate alpha rhythm appears to reflect a special state of “relaxed wakefulness”. Its maximum can be seen over the posterior parts of the brain, and in resting state conditions it is almost always greater when participants have closed compared to open eyes. Klimesch et al. (2007) developed an “inhibition-timing” hypothesis about alpha EEG activity, which entails that alpha power is inversely proportional to the activity of the underlying brain tissue. This hypothesis makes alpha activity extremely suitable for application in the field of sports, where sports performance is linked to neural efficiency, as argued above. If sports performance is linked to reduced brain activity, then this could be accompanied by an increase in EEG activity in the alpha band.

There is a lot of evidence supporting the positive relationship between sports performance and alpha activity, reviewed by several authors (e.g., Del Percio et al., 2011; Park et al., 2015). In much of this research, alpha activity is the dependent variable used as an index of the active inhibition of brain areas that are not required for the execution of a particular motor skill required for optimal sports performance. However, alpha activity has also been used as an independent variable. In this case, the goal is to teach athletes to gain control over alpha activity so that they can activate or inhibit it as the situation requires. Besides improving sports performance, alpha activity has long been associated with positive effects on relaxation, sleep, and well-being (e.g., Gruzelier, 2002).

In neurofeedback training (NFT), operant conditioning procedures are used to change brain rhythms, and it is then investigated how these changes relate to changes in performance. Landers et al. (1991) are usually credited as being the first to apply NFT for enhancing sports performance. They up-trained slow brain potentials over the left (“correct feedback”) and right (“incorrect feedback”) temporal cortex in archers and found that performance increased in the correct feedback group and decreased in the incorrect feedback group. Since then, there have been more applications of NFT research in sports. The reviews of Mirifar et al. (2017), and Rydzyk et al. (2023) indicate that NFT effectively improves the athletes’ performance in a specific sports task and/or in relevant underlying aspects of cognition and affect (Mirifar et al., 2017, p. 429).

There might be several reasons why NFT is slow to be adopted in the field of sports, despite the mostly positive effects on performance, relaxation, and well-being. One reason is that the traditional EEG setup with wired, gel-based electrodes does not fit well with the sporting environment. Secondly, NFT studies can take considerable time to complete. There are usually 10–20 training sessions to complete for each participant, there are training and control groups, so many NFT studies take half a year or more to complete. We (Van Boxtel et al., 2012) have initiated a program in an attempt to improve these drawbacks. A recording system was used in which the electrodes were moistened with tap water. The system was easier and quicker to set up than a standard laboratory recording system, but still needed supervision from a trained experimenter. With this system we trained alpha activity up in groups of five participants simultaneously. The feedback consisted of adapting music quality depending on the level of the brain rhythm to train. Using a double-blind between-subjects design, there were three conditions; alpha up (8–12 Hz), random beta up (different 4 Hz bands in the 13–30 Hz range), and no feedback (music only). The alpha up group showed a greater increase in alpha activity than either of the other two groups after the training, and especially on a follow-up measurement 6 weeks later, and that group also reported feeling more relaxed and comfortable compared to the other groups. In a subsequent study, we (Dekket et al., 2014) found that this approach was feasible in elite gymnasts. We found that alpha activity increased, and that this increase also resulted in an improvement of “being in shape”.

The goal of the present study is to take this approach a step further, and thereby to stimulate the adoption of NFT applications in elite sports. Recent technological advances have made EEG recordings much easier, and commercial, wireless, ‘dry’ EEG systems are now readily available. Because we wanted to work simultaneously in groups of people outside of a laboratory cabin, we asked two football (soccer) teams to participate. In this way, we also created a natural environment in which players can participate simultaneously during normal training hours at the club. In addition, elite clubs have already realized for a long time that success does not only depend on physical fitness and development of game tactics, but also on cognitive and psychological skills, such as attention, working memory, and executive functions. For this type of function, it is likely that NFT will be beneficial based on the neural efficiency hypothesis, in addition to having positive effects on relaxation, sleep, and well-being.

The participating clubs did not want to withhold a possible beneficial effect of NFT from any of their players. We therefore used a crossover design, in which there were three sessions intended to assess the effect of the NFT (assessment sessions A1–A3). Half of the group (A) received the NFT between A1 and A2, while the other half (B) received treatment as usual (TAU). After A2, the second group (B) received the NFT and the first group (A) received TAU.

The first question we wanted to address was whether simultaneous recordings were possible in groups, using a wireless EEG device connected to a smartphone. With many wireless devices connecting to smartphones, Bluetooth and WiFi interference is more than likely. We therefore started every assessment session with a resting state measurement of eyes open and eyes closed. For a good EEG measurement, we expected EEG spectra with decreasing power as a function of frequency, and a clear alpha peak superimposed on it around 10 Hz, which is greater under eyes closed than eyes open conditions.

Secondly, we expected alpha power to show alternating increases and decreases, as a function of the epoch within a training session. The approach in our previous work, as in the present study, was to alternate NFT epochs with cognitive tasks. This had two purposes. On the one hand, the participants were kept alert in this way without their attention flagging during the NFT periods in which they did nothing but listen to music. On the other hand, in this way we could immediately and easily collect data on cognitive processes. Keeping the order of alternating NFT and cognitive task epochs constant, we expected a ‘saw-tooth’ pattern of alpha activity with higher values in the NFT compared to the task periods.

Thirdly, we expected that alpha activity would increase as a result of the training. Given the cross-over design, this would result in an interaction between assessment session and group. Group A received the training between A1 and A2, and is therefore expected to show the largest increase in alpha activity between A1 and A2, not so much between A2 and A3. Group B received the training between A2 and A3, there is expected to show similar levels of alpha activity between A1 and A2, and an increase between A2 and A3. At A3, both groups are expected to show comparable levels of alpha activity.

Our fourth expectation was that the NFT resulted in a concomitant change in the performance on cognitive tasks and subjective questionnaires. The cognitive tasks that were used in the training to bring about alternation between NFT and cognitive epochs were task switching (executive functions), psychomotor vigilance (sustained attention), and mental rotation (mental representations). In the assessment sessions, the participants also performed a stop-signal task (response inhibition), attentional network task (alerting, orienting and executive attention), and N-back task (working memory). Taken together, these tasks were thought to provide a good overview of basic cognitive functions important for various aspects of the sports performance. In addition, we also collected subjective data about workload, mood, stress, sleep, and “being in shape”. Just like for the EEG data, we expected interactions between assessment sessions and groups with group A showing effects between A1 and A2 not between A2 and A3, and group B between A2 and A3 not between A1 and A2.

## Methods

### Participants

Two Dutch professional soccer teams participated in the study. The teams were from different clubs and were tested at different locations. One team consisted of 29 women competing at the highest national level, and the other team consisted of 19 males competing at the second national level. Most players were in their twenties, and 90% were right-handed. Ethical and legal considerations prevented us from collecting individual demographic data, motivated by the fact that some of them enjoyed national exposure and because the results of the study could not be shared with the club management at the individual level because this might have consequences for their future. Therefore we did not collect data which might allow them to be traced back to the individual.

Participation was voluntary and the participants were not paid. The study took place during their regular training hours. The data of 7 participants were discarded; 4 because they dropped out of the study or left the club, and 3 because there were technical issues, either in the assessment sessions or in a number of training sessions. The resulting group consisted of 26 females and 15 males, which for the present purposes were treated as a single group of 41 participants.

The study was approved by the Ethics Review Board of the Tilburg School of Social and Behavioral Sciences, ref. EC_TSB_RP770_2301_9306. Data storage and handling conformed to EU privacy regulations (GDPR).

### Apparatus

All EEG signals were collected with a BrainBit device (BrainBit Inc., Rancho Santa Margarita, CA, USA), It has the form of a flexible extension band with dry electrodes, an integrated electronic module and removable battery. The band is placed horizontally around the head above the ears, so that the electrodes are roughly located above 10–20 positions T3, T4, O1 and O2, all referred to the ground located on the forehead (roughly Fpz). Signals from these electrodes are sampled at a rate of 250 Hz and transmitted via Bluetooth LE to the receiving device (either a laptop or an iPhone—see below). We tested whether the device allowed us to record alpha activity in a similar way to a standard laboratory-style gel-based EEG recording system (Nexus-32F), and found it to be the case.

### Design

The study consisted of 20 training sessions for each participant, preferably one session per workday, thus spanning 4 weeks in total. Because of various commitments of the players, in practice the training sessions were spread over a period of about 6 weeks. The training sessions took place in a room at the clubs, in which up to 13 players were present at the same time, right before their field training started. If a player could not be present on a certain day, that training session was not repeated but skipped. Players with less than 15 training sessions were dropped from the study. On average, the players received 19.03 training sessions.

**Fig. 1:**
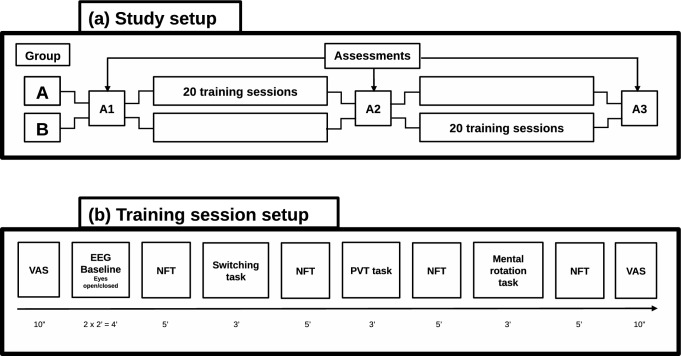
Study design and setup of a single training session

To assess the progress of the training, there were 3 assessment sessions, referred to as A1 to A3. Because the clubs wanted to offer the alpha neurofeedback training to all the players, we used a crossover design. A1 was always organized before the intervention started. In each team, half of the players received the intervention between A1 and A2 (group A), the other half between A2 and A3 (group B). A visual representation of the study design is presented in Fig. 1a.

### Assessment Sessions

The assessment sessions took place in a room at the club, containing a big table with a chair for each participant, making it possible to record the session for all participants at the same time. They received a standard 15’’ laptop with a screen refresh rate of 60 Hz, and an iPhone, also with a screen refresh rate of 60 Hz. The laptop contained home made software developed in Python to present three cognitive tasks: an N-back task, a stop-signal task, and an Attention Network Task (ANT), always in this order. In addition, the participants also performed one training session with the iPhone app (see below). The total duration of an assessment session was about one hour.

N-back Task (working memory). One out of a set of eight letters (B, F, K, H, M, Q, R, X) was presented centrally on the laptop screen for 500 ms, after which a fixation cross was presented for 1500 ms on average (1250–1750, rectangular distribution). On 50 out of 150 trials (33.3%), a letter was identical to the letter presented two letters before in the sequence. In that case, the participant had to press the ‘C’ button with their left hand. On the remaining 100 trials, this was not the case, and the participants had to press the ‘M’ button with their right hand. On 12.5% of the trials (‘lure’), the same letter was presented as the letter 1 or 3 letters before. In case of a correct response, the fixation cross turned green until the start of the next trial. On errors, the cross turned red.

Stop-Signal Task (response inhibition). A green arrow pointing left or right (50/50%) was presented at the center of the laptop screen for 800 ms. The participants were instructed to respond as quickly as possible with the index finger of the hand corresponding to the direction of the arrow by pressing either the ‘C’ or the ‘M’ key. On 50 of the 150 trials (33.3%), the arrow turned red for 100 ms, signaling the participants to withhold their response. The interval between the onset of the green arrow and the instant at which it turned red on a stop trial, was determined by a staircase tracking algorithm that started at 250 ms and increased or decreased by 50 ms depending on whether the response was withheld. After the response, a fixation cross was presented at the screen for an average duration of 1200 ms (950–1450 ms, rectangular distribution). Feedback was given when RT on a trial (excluding the first 5) exceeded the 75% percentile over the last 12 trials by showing the word “faster” on the screen. There was also feedback when the participant failed to stop at a stop signal: “STOP at red signal”.

ANT Task (attention). The target stimulus in this task was an array of five arrows pointing left or right (50/50%), which remained on the screen for 1700 ms. The participants were instructed to press the button ‘C’ or ‘M’ with their left or right hand, respectively, depending on the direction of the central arrow. The arrows flanking the central arrow could either point to the same direction as the central arrow (congruent, 33.3%) to the opposite direction (incongruent, 33.3%), or consist of a simple dash without arrowhead (neutral, 33.3%). The array of arrows was either presented above or below a central fixation cross (50/50%). At 400 ms before the target stimulus, a cue was presented for 100 ms in one of 4 conditions: no cue (cue equaled the fixation cross), central cue (fixation cross replaced by an asterisk), double cue (asterisks above and below the fixation cross), spatial cue (asterisk above or below the fixation cross, depending on where the target stimulus was going to be presented. In total, there were 4 cue types * 3 targets * 2 directions* 2 locations = 48 trial types repeated twice, totaling to 96 trials. The total trial length was 2200 ms, and the inter trial interval was 925 ms on average (325–1525 ms, rectangular distribution), during which the white fixation cross was presented, or a red cross in case of any error.

Questionnaires. The following subjective data were collected using visual-analog sliders on the laptop screen: Workload (NASA-TLX, Hart & Staveland, 1988), Mood (Profile of Mood States, POMS—short version, Shacham, 1983), Stress (Perceived Stress Scale, PSS-10, Cohen et al., 1983), Sleep (Athlete Sleep Screening Questionnaire, ASSQ, Driller et al., 2018), Being in shape (Dekker et al., 2014). The questionnaires were always presented in the same order.

### Training Sessions

A visual representation of the contents of a training session is shown in Fig. 1b. The participants used an iPhone with a specially designed app that guided them through the session. An iPhone was given to them in case they did not have a personal one. Each training session started and ended with a visual-analog slider by which the participants indicated their level of drowsiness, relaxation, and boredom (fixed order) on a scale from 0 to 100. The app then continued with the collection of 2 periods of 2 min resting-state EEG, in which the participants were sitting still with eyes open and eyes closed, respectively. This was followed by 4 NFT periods of 5 min each, which were alternated with 3 gamified cognitive tasks that lasted 3 min each; a switching task, a psychomotor vigilance task (PVT), and a mental rotation task, always in the same order. A single training session lasted about 45 min in total. The instructions for the alpha NFT periods was to just “sit back, relax, and listen to the music”. For the task periods, the participants were asked to execute the tasks as quickly as accurately as possible. It was possible that the players interacted during the training session, but a test leader was present who intervened when the players did not focus on the session.

EEG alpha training (NFT). The participants listened to their own favorite music that they selected before the start of the study, using earplugs or headphones. The music was passed through a high-pass filter that removed the low frequencies in the music based on the EEG alpha level of the brain signals. The lower the level of alpha activity, the more low frequencies were filtered out. This made the music sound distant and superficial if the alpha level was low, versus and full and rich when the alpha level was high, thus providing an intuitive feedback on the EEG alpha level based on the quality of the music. The procedure was similar to that described in our previous work (Van Boxtel et al., 2012). More specifically, five times per second (each 200 ms) a segment of the preceding 4 s of EEG data was filtered by fifth-order Butterworth filters at 1 Hz high-pass and 65 Hz low-pass, and a second-order notch filter at 50 Hz. To be usable for a feedback update, this segment should fulfil three criteria: (i) no clipping or overflow of the amplifier; (ii) peak-to-peak of at most 200 μV; (iii) the ratio between the line noise (49–51 Hz) and EEG power (4–30 Hz) should be smaller than 1.0.for each electrode. The relative alpha power was then calculated as the sum of the power in the alpha band (7.7–12.3 Hz), divided by the sum of the power in the beta band total power (14.7–25.3 Hz). If both electrodes showed a good epoch of 4 s, then the average of the two electrodes was used. If only one electrode yielded a good epoch, then only that channel was used. If neither electrode resulted in a good epoch, then no feedback update was given. The relative alpha measure was filtered by a first-order IIR filter with a time constant of 4 s. In this way, the speed of the changes in the feedback was smoothed somewhat, and did not go back and forth too quickly. The feedback measure was used to drive the cut-off frequency of a first-order high-pass filter built into the audio path of the music played through the headphones. This cut-off frequency equaled 2 Hz if the current alpha level was greater than the maximum alpha level for the previous part, and 1500 Hz if the current alpha level was lower than the minimum alpha level for the previous part. For intermediate levels, a linear interpolation was done in such a way that the cut-off frequency was high for low current alpha levels and low for high alpha levels.

Switching Task. The gamified switching task, called “deluge of dice” is played on the iPhone. A die containing 1 to 6 dots is displayed either at the top or at the bottom of the screen (50/50%, 30% switches, 15% top–bottom and 15% bottom-top). The die could appear outlined or solid (50/50%). If it appeared on the top, the participants had to ignore the number of dots and respond with the left or the right hand, according to whether the die was outlined or filled, respectively. If it appeared on the bottom, they had to ignore the form and respond to the number of dots; left for 1 to 3 dots, right for 4 to 6 dots. A die was presented every 2000 ms on average (1750–2250 ms, rectangular distribution), and stayed on the screen during the whole trial period. When a response was given, the color of the dice turned green or red, depending on whether the response was correct or not. The task was stopped after 3 min, during which approximately ninety trials were administered on average.

PVT Task. The gamified psychomotor vigilance task, called “react-o-matic”, was a simple reaction time task focused on response speed. A white outline of a circle filled with black (background) was shown on the screen, which required to be responded to as soon as its center became filled with white. At that same instant a black outlined circle started to grow from the inside out, on the now white background, which stopped as soon as a button at the bottom of the screen was pressed. This gave intuitive feedback about response speed. The actual reaction time value in milliseconds was also displayed on the screen as soon the response was given, or the words “too early” or “too slow” on premature reactions and timeouts, respectively. Half of the trials were presented with a short interval (2–5 s, rectangular distribution); the other half with a long interval (5–10 s, linearly descending distribution). Again, this task lasted 3 min, during which an average total of 30 trials were administered.

Mental Rotation. The gamified mental rotation task was called “typo trap”, and involved the display of one out of four numbers, 2, 4, 5, or 7, for a duration of 300 ms. The numbers were randomly rotated around the Y-axis of a coordinate system, in steps of 30 degrees. On half of the trials, the letter was mirrored, and this was integrated with the rotation. The task of the participants was to indicate whether the number was mirrored (press left button) or not (press right button). The degree of rotation was to be ignored. If a correct response was given, the number reappeared in the color green, otherwise it reappeared in red. In the 3 min task period, an average total of 96 trials were administered at a pace of 1875 ms (1625–2125 ms, rectangular distribution).

### Data Processing

EEG data. The EEG data were subjected to extensive data preprocessing. This was needed because of the large between- and within-subject variability caused by the field conditions under which the experiment was carried out. We used a Welch spectrum with segments of 4 s and 75% overlap. Before the transformation into the frequency domain, we first computed, for each segment and electrode montage, the difference between the maximum and minimum EEG amplitude in that segment. All segments for a specific task in each assessment session and for each electrode montage were then modeled by an exponential distribution, and only those segments within the 95% confidence interval were selected for further processing and transformation into the frequency domain.

This procedure resulted in loss of data, ranging across participants from 12 to 68%. On average, there was 32% data loss (median 31%), slightly more in the left electrode montage T3–O1 then in the right T4–O2, 33% vs 31%. The data loss consisted of lost data packages in the Bluetooth transmission between the EEG device and the app or laptop, as well as discarded segments because of movement-related or other artifacts detected.

The accepted 4-s segments were band-pass filtered using a first-order Butterworth filter, tapered using a Hamming window, and then transformed into the frequency domain by FFT. The transformed segments were then averaged to obtain the Welch spectrum. The average power (log10 transformed squared magnitude) in the alpha band (7.7–12.3 Hz) was normalized by the power in the beta band (14.7–25.3 Hz) to obtain more stable spectra. This was done because we wanted to remain as close as possible to the way the neurofeedback training was administered. We refer to this activity as ‘normalized alpha’. To disentangle alpha from beta activity, we used Principal Components Analysis (PCA) on the frequency spectra using the covariance matrix and Varimax rotation to simple structure. The Scree plot was used to determine how many components to extract, and the component scores for those components were then analyzed in the same way as the raw power values. PCA was used instead of further analyses of raw alpha and beta power, because it indicates which frequency bands are best used based on the variance in the experimental data, rather than using predefined bands, and has the additional advantage of normalizing the power.

Cognitive tasks. An exGaussian model was used to fit reaction times to the cognitive tasks. It consists of a Gaussian distribution with parameters μ and σ, representing the mean and standard-deviation of a normal distribution, overlapped with an exponential distribution with parameter τ, which models the variability in the right-hand tail of the RT distribution.

### Statistical Analysis

All analyses were done by linear mixed-effects models with restricted maximum likelihood estimation, using the Satterthwaite method to estimate the degrees of freedom needed for calculating p-values. Focus was on the interaction between Assessment Session and Group. In case of statistically reliable interactions, simple effects were calculated for further exploration.

## Results

### EEG Data

**Fig. 2:**
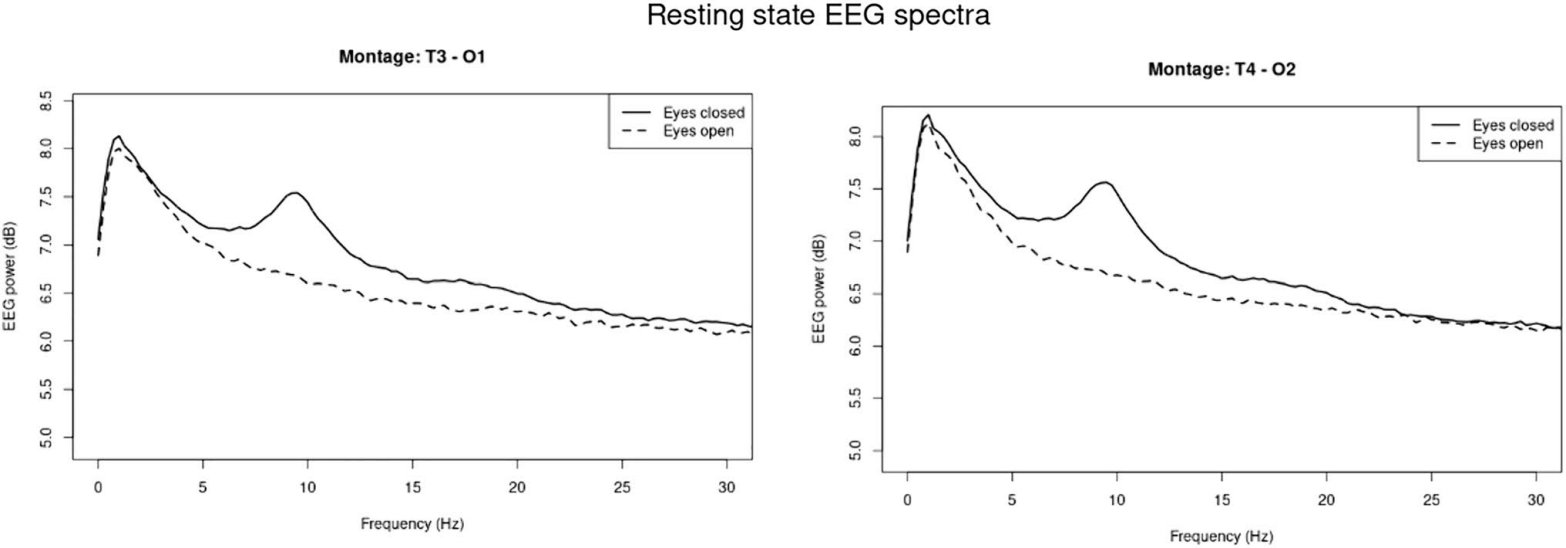
Spectra of eyes open and closed resting state epochs at session A1

Resting state. Averaged spectra in eyes open and eyes closed resting state conditions in the assessment session A1, are presented in Fig. 2, separately for the two montages T3–O1 and T4–O2. As can be seen in Fig. 2, the spectra have the characteristic property that power diminishes with increasing frequency (1 / f ratio). A prominent alpha power peak is also clearly present in the 8–12 Hz range, which is also characteristic for EEG power spectra. As expected, normalized alpha power was greater in eyes closed compared to eyes open conditions (F(1, 111.22) = 160.91, p < 0.001), and did not differ between the montages (F(1, 110.50) = 0.01). The Scree plot of the PCA on these spectra suggested extracting and rotating 3 components, explaining 92% of the variance. The second extracted component, which explained 20% of the variance, showed loading with a maximum around 10 Hz, and its scores showed that it was greater in the eyes closed compared to the eyes open situation (F(1, 113.05) = 8.93, p < 0.01). It could therefore be identified as an “alpha” component. The other two rotated components did not show any effects. The results of the PCA thus corroborated the analyses on the raw data.

Taken together, these results show that it is possible to reliably record EEG using a wireless device with a Bluetooth connection, from up to 13 participants at the same time. The normalized alpha power in the eyes closed resting state condition was greater than the alpha power recorded during eyes open.

**Fig. 3:**
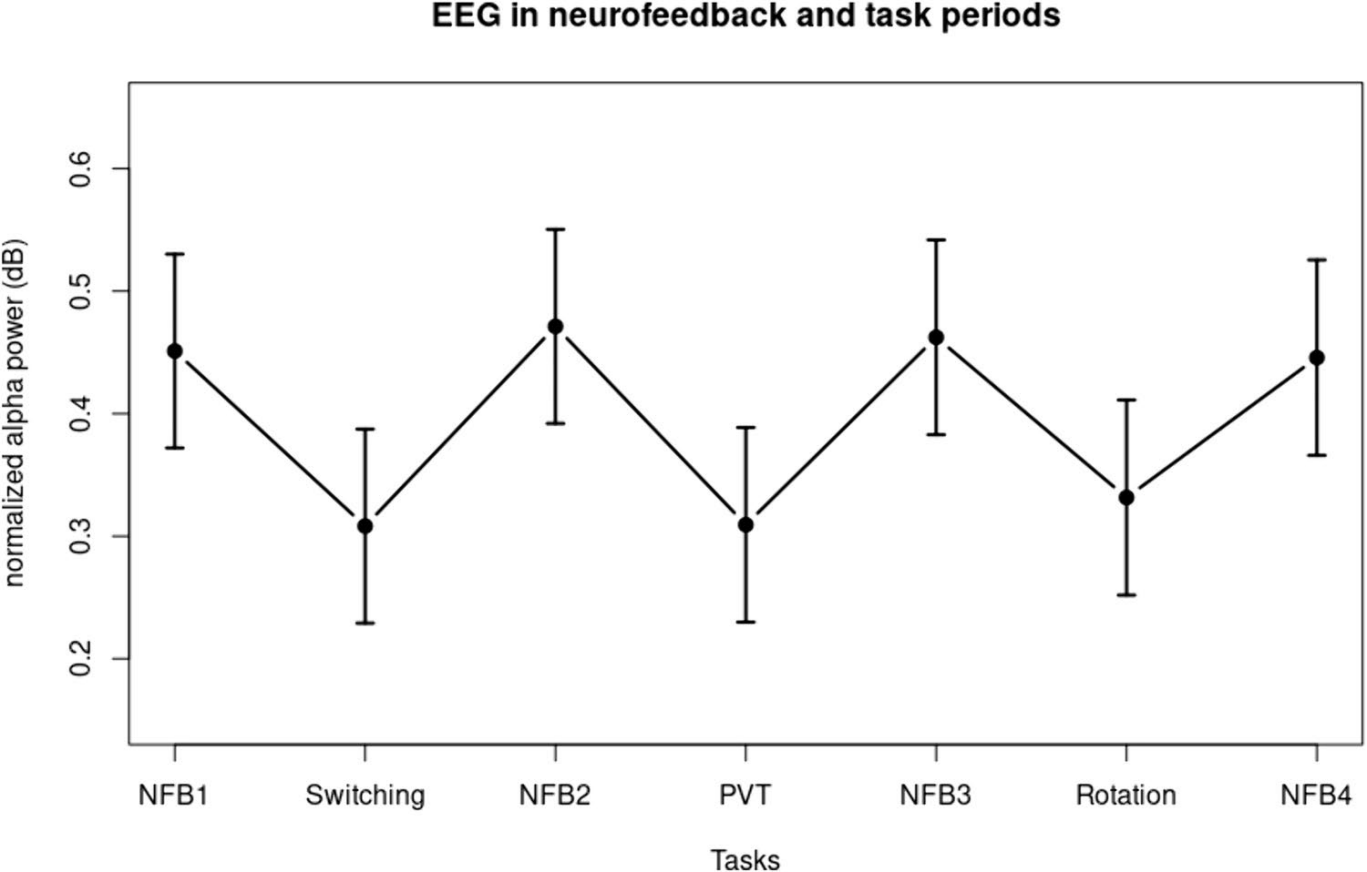
EEG in neurofeedback and task periods, averaged over assessment sessions and montages

Neurofeedback and task periods. Normalized alpha power in the periods of neurofeedback and cognitive tasks, averaged over the two montages and the three assessment sessions, are depicted in Fig. 3. It can be seen that it was greater in the neurofeedback periods compared to the task periods (F(1, 1344.3) = 124.54, p < 0.001). The PCA on the raw spectra resulted in the extraction of separable alpha and beta components, explaining 21% and 52% of the total variance, respectively (see Fig. 5). Analyses of the scores of these extracted components revealed that beta power was greater in the task periods compared to the neurofeedback periods (F(1, 1343.6) = 130.77, p < 0.001), but alpha power was not statistically separable between neurofeedback and task periods ((1, 1344.4) = 0.15, n.s.). In other words, the effect observed in Fig. 3 was due to beta power, not alpha power.

**Fig. 4:**
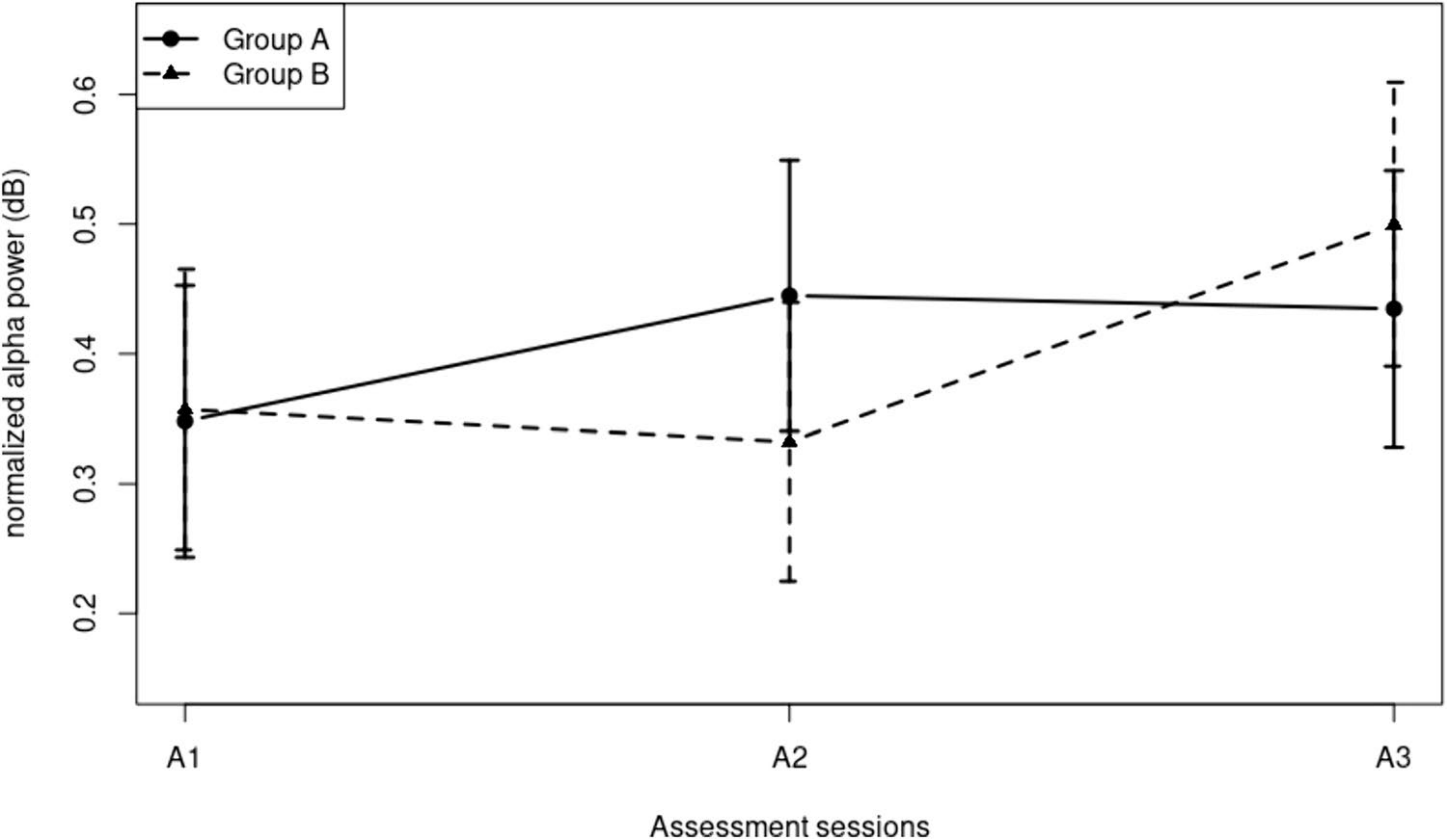
Alpha power averaged over montages, by assessment sessions, separately for the groups

Turning now to the changes of EEG power over the three assessment sessions, we found that normalized alpha power increased over the sessions by 34.3%, from 0.35 in A1 to 0.47 in A3 (F(2, 1349.77) = 22.84, p < 0.001). Although there was no overall difference between the two groups (F(1, 39.17) = 0.03, n.s.), there was an interaction between assessment sessions and groups (F(2, 1349.77) = 15.88, p < 0.001). The estimated marginal means of this interaction are displayed in Fig. 4. Simple effects confirmed the pattern that can be observed in the figure that normalized alpha power increased as a result of the neurofeedback training, that is, for group A between A1 and A2 (F(1, 503.99) = 27.16, p < 0.001) not between A2 and A3 (F(1, 451.42) = 0.46, n.s.), and for group B between A2 and A3 (F(1, 426.32) = 48.45, p < 0.001) not between A1 and A2 (F(1, 467.36) = 1.63, n.s.).

**Fig. 5:**
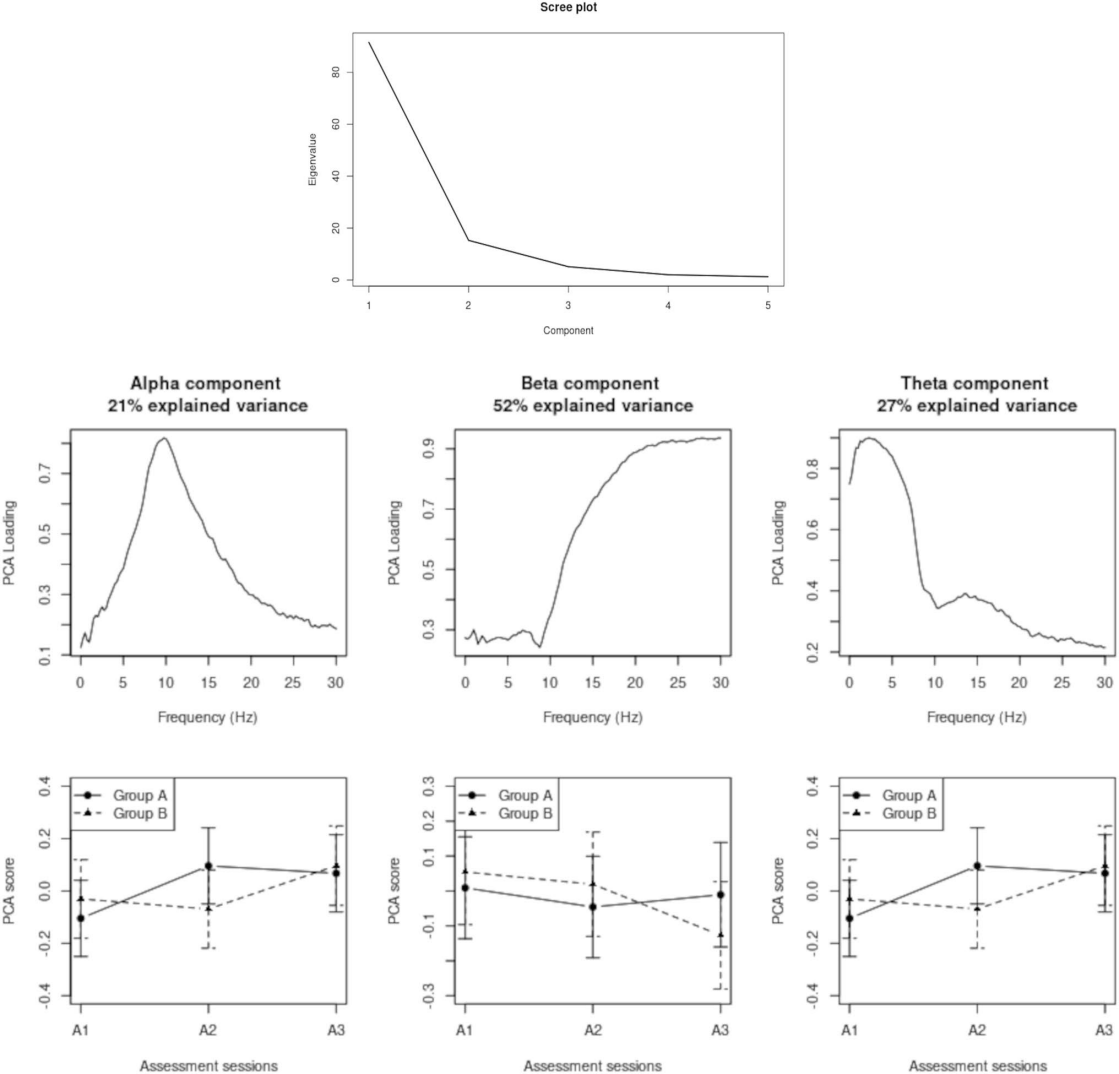
PCA component loadings for alpha, beta, and theta activity, and their effect on the component scores

The results of the PCA (Fig. 5) confirmed these findings. The Scree plot suggested extracting 3 components, together explaining 92% of the variance. For the alpha component (21% of variance), there was an overall increase over assessment sessions (F(2, 1348.17) = 25.33, p < 0.001). The increase occurred between A1 and A2 for group A, and between A2 and A3 for group B (F(2, 1348.17) = 22.34, p < 0.001). The beta component (52% of variance) decreased over assessment sessions (F(2, 1352.76) = 6.13, p < 0.01); the decrease occurred between A2 and A3 for group B not for group A (F(2, 1352.76) = 5.80, p < 0.01). The PCA also resulted in component with high loading in the theta range, explaining 27% of the variance. The analysis of the scores of this component resembled that of the alpha component, with an overall increase over assessment sessions (F(2, 1353,92) = 25.66, p < 0.001), which occurred between A1 and A2 for group A and between A2 and A3 for group B (F(2, 1353.92) = 4.45, p < 0.05).

### Behavioral Data

Cognitive tasks. An overview of the results obtained from the cognitive tasks is presented in Table 1. The most important effect for all tasks was the interaction between Group and Assessment Session, which is highlighted in the table. Plots of the estimated marginal means involved in this interaction is presented as supplementary information S1. It can be seen in the table that performance was fast and accurate, and that there were clear learning effects (main effects of assessment sessions). The interactions between groups and assessment sessions were observed for the switching and the mental rotation tasks, and the means involved in these effects showed that learning was indeed enhanced between A1 and A2 for group A, and between A2 and A3 for group B. Importantly, the estimated marginal means involved in these effects (S1) showed a similar pattern as normalized alpha activity (Fig. 4) and the PCA alpha component, not the PCA beta component (Fig. 5).

**Table 1:** Results of performance on cognitive tasks

(a) N-back task								
	RT μ		RT σ		RT τ		Prop. correct	
	*df*	*F*	*df*	*F*	*df*	*F*	*df*	*F*
Session	2, 62.700	4.6592*	2, 65.414	6.2222**	2, 63.108	3.7897*	2, 54.811	7.6989**
Group	1, 37.684	1.6834	1, 38.088	0.6081	1, 35.826	0.0918	1, 29.830	1.1845
Session* Group	2, 62.700	1.0550	2, 65.414	1.9360	2, 63.108	0.1677	2, 54.811	1.2767

(b) Stop-signal task								
	Go RT μ		Go RT σ		Go RT τ		SSRT	
	*df*	*F*	*df*	*F*	*df*	*F*	*df*	*F*
Session	2, 64.496	5.8137***	2, 66.803	7.8580***	2, 60.490	12.8062***	2, 55.436	33.710*
Group	1, 38.791	20.5501***	1, 39.495	7.0759*	1, 30.024	6.2112*	1, 28.389	0.0216
Session* Group	2, 64.496	0.7594	2, 66.803	1.0272	2, 60.490	0.1884	2, 55.436	2.5278

(c) ANT task								
	RT μ		RT σ		RT τ		Prop. correct	
	*df*	F	df	F	df	F	df	F
Session	2, 294.497	4.9744**	2, 294.080	0.3649	2, 295.200	0.6383	2, 294.799	10.3960***
Group	1, 37.267	0.8580	1, 36.106	0.0041	1, 36.221	0.0078	1, 39.152	3.3395
Target	2, 289.198	21.5396***	2, 287.946	0.8071	2, 288.149	0.6444	2, 291.107	258.5536***
Session * Group	2, 294.497	1.0648	2, 294.080	0.6253	2, 295.200	1.2586	2, 294.799	1.5902
Session * Target	2, 289.198	0.2653	4, 287.946	0.1573	4, 288.149	0.3788	4, 291.107	5.0692***
Group * Target	2, 289.198	0.0622	2, 287.946	0.2568	2, 288.149	0.2055	2, 291.107	7.0791***
Session * Group * Target	4, 289.198	0.9478	4, 287.946	0.3217	4, 288.149	0.7813	4, 291.107	0.4749

(d) Switching task										
	RT μ		RT σ		RT τ		Prop. correct		Swiching costs	
	*df*	*F*	*df*	*F*	*df*	*F*	*df*	*F*	*df*	*F*
Session	2, 370.62	66.0476***	2, 373.43	45.9709***	2, 376.81	37.5947***	2, 375.43	11.6701***	2, 380.63	2.5636
Group	1, 34.14	1.2220	1, 34.21	0.8484	1, 35.02	6.1285*	1, 35.51	3.8943	1, 32.91	1.2846
	1, 368.10	331.4338***	1, 368.20	33.1599***	1, 369.13	12.0706***	1, 369.53	21.2978***		
Session * Group	2, 370.62	12.0847***	2, 373.43	8.2721***	2, 376.81	15.2115***	2, 375.43	34.5260***	2, 380.63	1.0431
Session * Switch	2, 368.10	0.6908	2, 368.20	1.9718	2, 369.13	0.9582	2, 369.53	3.4716		
Group * Switch	1, 368.10	1.2405	1, 368.20	5.1042*	1, 369.13	0.1743	1, 369.53	0.3480		
Session * Group * Switch	2, 368.10	0.4775	2, 368.20	2.5510	2, 369.13	0.3317	2, 369.53	0.5079		

(e) Psychomotor vigilance task								
	RT μ		RT σ		RT τ		Prop. correct	
	*df*	*F*	*df*	*F*	*df*	*F*	*df*	*F*
Session	2, 301.550	0.3517	2, 314.857	3.1880*	2, 317.95	2.6845	2, 65.606	3.7857*
Group	1, 38.939	1.2413	1, 27.305	4.6336*	1, 33.49	8.1684**	1, 33.945	1.1632
Session* Group	2, 301.550	0.8615	2, 314.857	1.1405	2, 317.95	1.2703	2, 65.606	1.9489

(f) Mental rotation task								
	RT μ		RT σ		RT τ		Prop. correct	
	*df*	*F*	*df*	*F*	*df*	*F*	*df*	*F*
Session	2, 176.842	2.3181	2, 173.606	5.8988**	2, 175.51	4.1685*	2, 64.949	15.6035***
Group	1, 36.684	0.8826	1, 29.839	3.7076	1, 29.51	8.5135**	1, 34.862	2.9488
Session* Group	2, 176.842	2.6557	2, 173.606	2.1072	2, 175.51	2.2231	2, 64.949	9.5638***

*p < 0.05, **p < 0.01, ***p < 0.001

Questionnaires. An overview of the results obtained from the subjective questionnaires is presented in Table 2. The most important effect for all questionnaires was the interaction between Group and Assessment Session, which is highlighted in the table. Plots of the estimated marginal means involved in this interaction is presented in the supplementary information S2. There were no main effects of assessment sessions and groups. Interactions between these two factors were observed for the sleep duration question from the ASSQ, and for the ‘feeling on control’ and ‘flow’ variables of the Being in Shape questionnaire.

**Table 2:** Result of subjective questionnaires

	Session		Group		Session * Group	
	*df*	*F*	*df*	*F*	*df*	*F*
(a) Workload (NASA-TLX)						
Mental Demand	2, 73.053	0.4609	1, 39.787	0.5268	2, 73.053	0.4552
Physical Demand	2, 71.559	2.4707	1, 38.524	1.4259	2, 71.559	1.6325
Temporal Demand	2, 72.562	0.6899	1, 39.293	0.0023	2, 72.562	0.4053
Performance	2, 75.191	0.3407	1, 40.346	1.5761	2, 75.191	1.2586
Effort	2, 74.016	4.0898*	1,40.288	3.3624	2, 74.016	0.0299
Frustration	2, 72.331	0.5586	1, 39.174	0.1406	2, 72.331	0.3071
(b) Mood (POMS)						
Tension/Anxiety	2, 73.286	0.6755	1, 39.683	0.7638	2, 73.286	0.3560
Anger/Hostility	2, 74.056	0.2580	1, 40.251	0.2284	2, 74.056	0.0992
Vigor/Activity	2, 72.425	2.7084	1, 38.525	0.2140	2, 72.425	1.3437
Fatigue/Inertia	2, 74.090	5.8872**	1, 39.227	1.9509	2, 74.090	1.8551
Depression/Dejection	2, 73.008	0.2216	1, 39.283	0.1671	2, 73.008	1.2117
(c) Stress (PSS-10)						
Perceived helpnessness	2, 72.436	0.5195	1, 39.607	0.0050	2, 72.436	0.8018
Lack of self-efficacy	2, 72.510	1.0156	1, 39.200	0.4261	2, 72.510	0.5153
(d) Sleep (ASSQ)						
Sleep duration	2, 72.733	1.1716	1, 39.617	0.0005	2, 72.733	4.7522*
Time to fall asleep	2, 71.703	1.6051	1, 39.259	0.1931	2, 71.703	2.0861
Sleep satisfaction	2, 73.903	1.4633	1, 39.869	0.3940	2, 73.903	0.0705
(f) Being in shape						
Physical shape	2, 74.058	0.0633	1, 39.519	0.3661	2, 74.058	1.5271
Ability to recover	2, 73.230	0.3892	1, 39.569	1.1374	2, 73.230	1.6739
Feeling of control	2, 72.052	1.2522	1, 38.721	0.0014	2, 72.052	3.3413*
Mental balance	2, 74.270	2.5148	1, 39.831	0.1535	2, 74.270	1.4458
Confidence	2, 71.821	0.4413	1, 39.016	0.0168	2, 71.821	1.2661
Committment	2, 71.765	1.6611	1, 37.745	0.0002	2, 71.765	0.1929
Focus	2, 71.549	4.2797*	1, 37.676	0.1074	2, 71.549	0.0958
I rritation	2, 73.887	0.1098	1, 39.553	1.3433	2, 73.887	0.8397
External factors affecting performance	2, 74.335	0.4917	1, 39.987	0.6506	2, 74.335	0.0991
Mental shape	2, 74.139	0.7572	1, 40.065	0.6730	2, 74.139	1.4998
Ability to suppress distraction	2, 73.898	1.2805	1, 38.826	0.2433	2, 73.898	0.4439
Flow	2, 73.506	0.6051	1, 40.094	1.1303	2, 73.506	4.1830*
Resilience to stress	2, 72.016	1.8746	1, 38.514	0.1901	2, 72.016	0.0047
Ability to follow instructions	2, 71.865	3.8924*	1, 38.625	3.9460	2, 71.865	0.9186
Ability to collaborate with others	2, 70.367	0.5452	1, 36.667	0.3671	2, 70.367	0.1238
(f) Visual-analog scale, post-pre						
Drowsiness	2, 72.695	0.8315	1, 39.969	3.7039	2, 72.695	1.6026
Relaxation	2, 68.082	0.4965	1, 37.735	0.7611	2, 68.082	0.8710
Boredom	2, 72.337	1.5537	1, 39.716	0.4127	2, 72.337	0.5316

## Discussion

The aim of this study was to investigate if EEG alpha activity could be increased in elite athletes who, in a groups of participants simultaneously received neurofeedback training with a modern wireless EEG recording device. In addition, we wanted to investigate if the increase in EEG alpha activity would improve cognitive performance as well as subjective experience of well-being, such as mood and sleep.

The first question was whether it was possible to reliably record EEG alpha activity simultaneously in groups of participants using a wireless device. This turned out to be possible. We also showed that it was possible to do so in groups of up to 13 people at the same time. There was approximately 30% data loss due to the simultaneous recordings (lost data packages) and the field conditions outside of a standard EEG laboratory. This was higher than normally observed in a standard laboratory using gel-based wired EEG equipment, where 10–15% data loss is usually expected. It is good to realize that these types of experiments will result in a higher than expected data loss, but otherwise excluding data segments with lost data packages and other artifacts did result in EEG power spectra as they can usually be observed. The spectra had the characteristic feature that power decreases with increasing frequency. The spectra also exhibited a clear alpha power peak in the 8–12 Hz range, which was greater with eyes closed compared to eyes open. These two phenomena together are the defining characteristics of EEG spectra, and it is therefore safe to conclude that our setup with simultaneous recordings using a wireless device was successful. In addition, the result of the PCA, corroborated and strengthened this conclusion.

We used a specific device for our study, but it can be expected that similar devices on the market will produce similar results. Websites of companies that sell those products often do show EEG spectra similar to what we reported here, but to our knowledge, we are the first to demonstrate the use of such equipment simultaneously in larger groups. This makes it possible to do this type of research in teams, which is advantageous for team spirit, and also reduces research time considerably, because the recordings do not need to be done serially.

The second question concerned the nature of our training setup with alternating epochs of neurofeedback training and cognitive tasks. The setup was chosen to keep the participants alert by not using too long periods of sitting still and listening to music, while at the same time collecting data about cognitive performance during various tasks. We predicted that neurofeedback training periods would show greater alpha activity compared to epochs during which the participants performed cognitive tasks. In addition, due to the fixed alternating order, we predicted that a ‘saw-tooth’ pattern would be visible. The first impression was that this indeed seemed to be the case, based on our definition of alpha activity. Both the saw-tooth pattern was found, and alpha activity in the average of all neurofeedback training epochs, compared to the average of the cognitive task epochs, was also different in a statistically reliable way.

However, our definition of alpha activity was based on alpha power normalized by beta power. This was done to make the spectra more comparable between participants and sessions, but also begged the question whether the saw-tooth pattern was the result of changes in alpha activity or of mirrored changes in beta activity. Alpha activity was expected to be high in neurofeedback epochs and low in task epochs, but the reverse could be expected for beta activity. Taking the alpha / beta ratio could then be the result of changes in alpha, changes in beta, or a combination of the two. We tried to disentangle the two using PCA, and found a clear saw-tooth pattern for the component reflecting beta activity, and a much less clear pattern for the component interpreted as reflecting alpha activity. The scores associated with the extracted component with loading in the beta range were greater for the task periods compared to the neurofeedback training periods, whereas the alpha component’s scores did not differ between epochs. Therefore, the conclusion seems warranted that the saw-tooth pattern was mainly the result of changes in beta not alpha activity.

The third hypothesis concerned the question whether alpha activity would increase as a result of the training. Given the cross-over design that was used, this would imply that alpha activity would always increase between the assessment sessions during which the training was administered, not between the assessment sessions during which there was no training (treatment as usual). This was exactly what we found. For the group that received the training between the first and second assessment sessions, the increase in alpha activity occurred between those two sessions not between the second and third session. For the group that received the training between the second and third assessment sessions, there was no increase between the first and second sessions, but between the second and third. Both groups reached the same level of alpha activity at the third assessment session. The results of the PCA alpha component exactly showed the same pattern of results. Using two different methods of analysis that arrive at the same conclusions enhances the validity of those conclusions. Interestingly, the PCA beta component showed decreases in the same training periods in which the alpha component increased. Together these components produce the results found in global alpha activity scores as defined. In addition, it can also be concluded that the neurofeedback training increased alpha activity while at the same time decreasing beta activity.

The increase in alpha activity was approximately one-third of baseline levels. Given the present design, it is unsure whether this increase resulted from the neurofeedback training itself, from sitting still and listening to music during the training, from both, or from other aspects of the study. The reason is that we did not include control conditions in the study setup, because the clubs wanted to offer the training to all players without making a group who received no training or another intervention. However, in our previous work (Van Boxtel et al., 2012) we used three groups. One group received genuine alpha activity training, much the same as in the present work. Another group received beta activity training in 4 Hz bands that were different in every training session, and, interestingly, a third group just sat still and listened to their favorite music. The increase in alpha activity in the real alpha training group was very similar to what we found in the present work (33%), but the increase in the music only group was quite small, about 5%. This shows that the increase was at least partly due to the neurofeedback training.

It is difficult to compare the present findings to other studies, because of the many differences between studies in electrode location, training protocol, control groups, etc. Several studies suggest that our findings are reasonably comparable to what others have found, though. For instance, Cho et al (2008) reported an increase of up to 50% in alpha activity level over 11 training sessions of 17.5 min each. More recently, Su et al. (2021) reported an increase of about 40% over 12 sessions of 6 min each, and Shen et al. (2023) found an increase of 13% over 5 sessions of 25 min each. All in all, it seems safe to conclude that the present setup using simultaneous auditory neurofeedback training in groups of elite athletes using a wireless EEG device, was successful in increasing EEG alpha activity.

The next question then is if the increase in EEG alpha activity has effects on the cognitive behavior and sense of well-being of the athletes. Judging from the subjective data as measured by the questionnaires and slider scales, these effects are limited, if they exist at all. Self-reported sleep duration and fatigue increased slightly, and the amount of effort invested in the tasks decreased, but none of these effects could be related to the phases of the training. Therefore these were just overall effects of the training program as a whole. A possibly interesting effect that was specific for the groups and the assessment session, were the questions about “feeling in control” and “being in a flow” of the Being in Shape questionnaire, but these interactions were not entirely consistent and the psychometric properties of that questionnaire are unknown. The visual sliders used before and after each individual training session resulted in more consistent findings. It appeared that the athletes found the training to be relaxing, but also not very challenging, perhaps reflecting the fact that the training sessions were organized just before their regular training when the players were eager to go out onto the pitch. Moreover, relating subjective data to neurofeedback training is often found to be very difficult because of the fact that people interpret questions in different ways and there is little standardization.

As to the cognitive tasks, performance improved over the assessment sessions. For most tasks, mean and variability of response times decreased and accuracy increased as a result of training. For the relatively basic cognitive processes, such as working memory, psychomotor vigilance, response inhibition, and attention, the training effects did not depend on the instant at which the intervention was given. By contrast, the higher cognitive processes of task switching and mental rotation did show those effects. It is striking that these more difficult tasks show effects of neurofeedback training in this group, because these are preeminently cognitive processes that are necessary to achieve good performance on the field in a team sport. It is quite difficult to interpret the current results in terms of the existing literature in this area, especially specifically for sports, because there are large differences in the tasks used, the brain rhythms trained, the electrode positions, etc. It is very well possible that neurofeedback training outside of the alpha range works well for improving response speed on relatively simple cognitive tasks (e.g., Brito et al., 2022), and that higher cognitive processes mostly benefit from enhancing alpha rhythms, as suggested by the present work (see also Takabatake et al., 2021). Here we did find that the effects in the switching and mental rotation tasks resembled the effects of alpha activity more than beta activity, but perhaps the relationship is too uncertain to draw definitive conclusions. This is an interesting line of research for future work, in which individual differences in trainability of both brain rhythms and cognitive tasks can be taken into account.

Taken together, the results of this study show that it is feasible to conduct neurofeedback training in groups as opposed to individually, using a wireless device which is easy to set up. This implies that neurofeedback studies can be made much more (cost) efficient compared to individual training sessions, which is likely to stimulate the application of this type of research for team sports.

## Supplementary Information

Below is the link to the electronic supplementary material.

Supplementary file1 (PDF 762 KB)

Supplementary file2 (PDF 937 KB)

## Data Availability

The participants of this study did not give written consent for their data to be shared publicly, so due to the sensitive nature of the research supporting data is not available.
