# Supplementary material for: Alpha Neurofeedback Training in Elite Soccer Players Trained in Groups
Source: Appl Psychophysiol Biofeedback. 2024 Aug 10;49(4):589–602. doi: 10.1007/s10484-024-09654-1 (PMC11588833; doi:10.1007/s10484-024-09654-1)
Supplement: Supplementary file 1 — Supplementary file1 (PDF 762 KB) [file 10484_2024_9654_MOESM1_ESM.pdf]

N-back task

	RT **μ**		RT **σ**		RT **τ**		prop. correct	
	df	*F*	*df*	*F*	*df*	*F*	*df*	*F*
Session	2, 62.700	4.6592 *	2, 65.414	6.2222 **	2, 63.108	3.7897 *	2, 54.811	7.6989 **
Group	1, 37.684	1.6834	1, 38.088	0.6081	1, 35.826	0.0918	1, 29.830	1.1845
Session* Group	2, 62.700	1.0550	2, 65.414	1.9360	2, 63.108	0.1677	2, 54.811	1.2767

*p < 0.05 **p < 0.01 ***p < 0.001

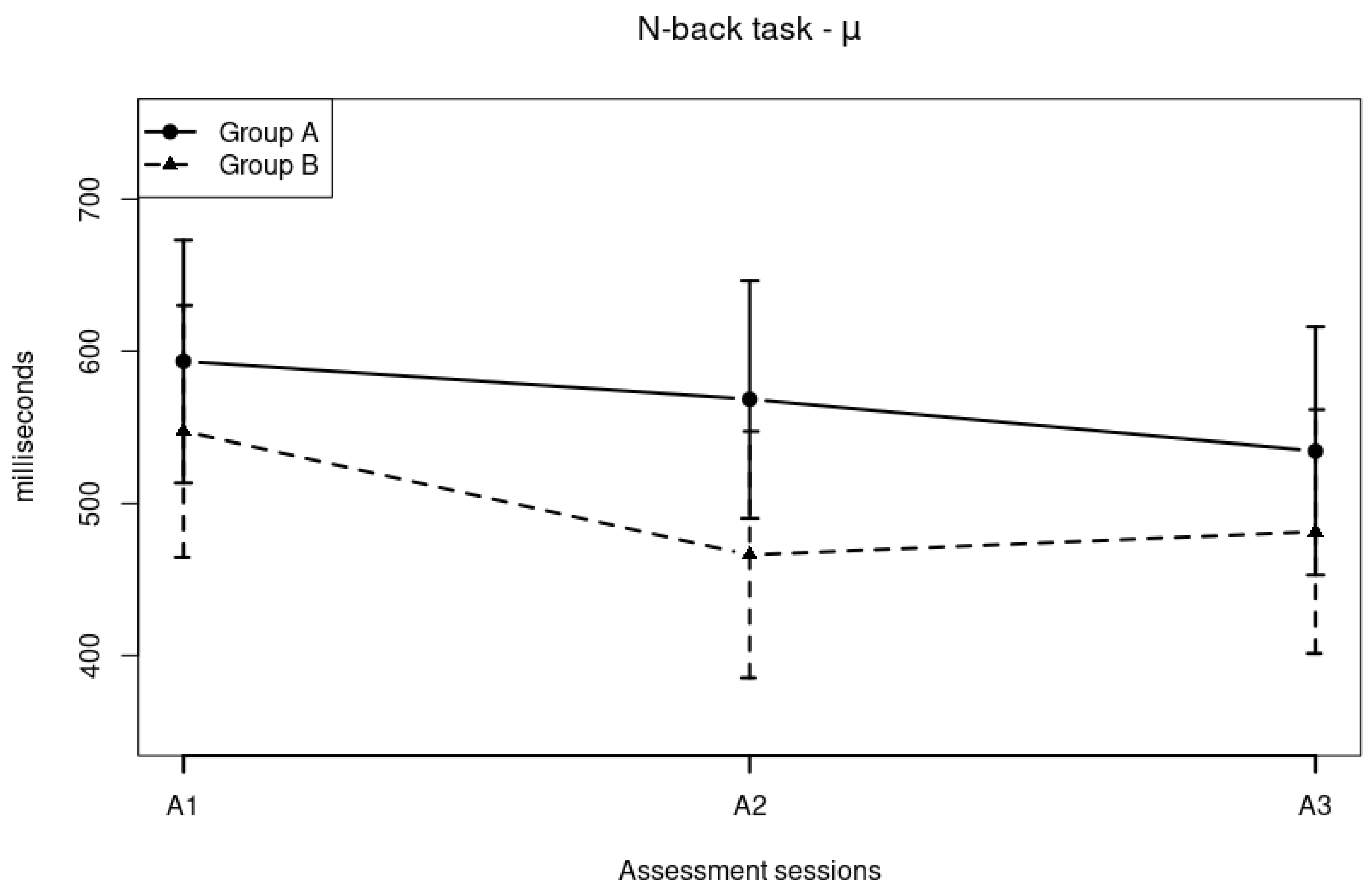

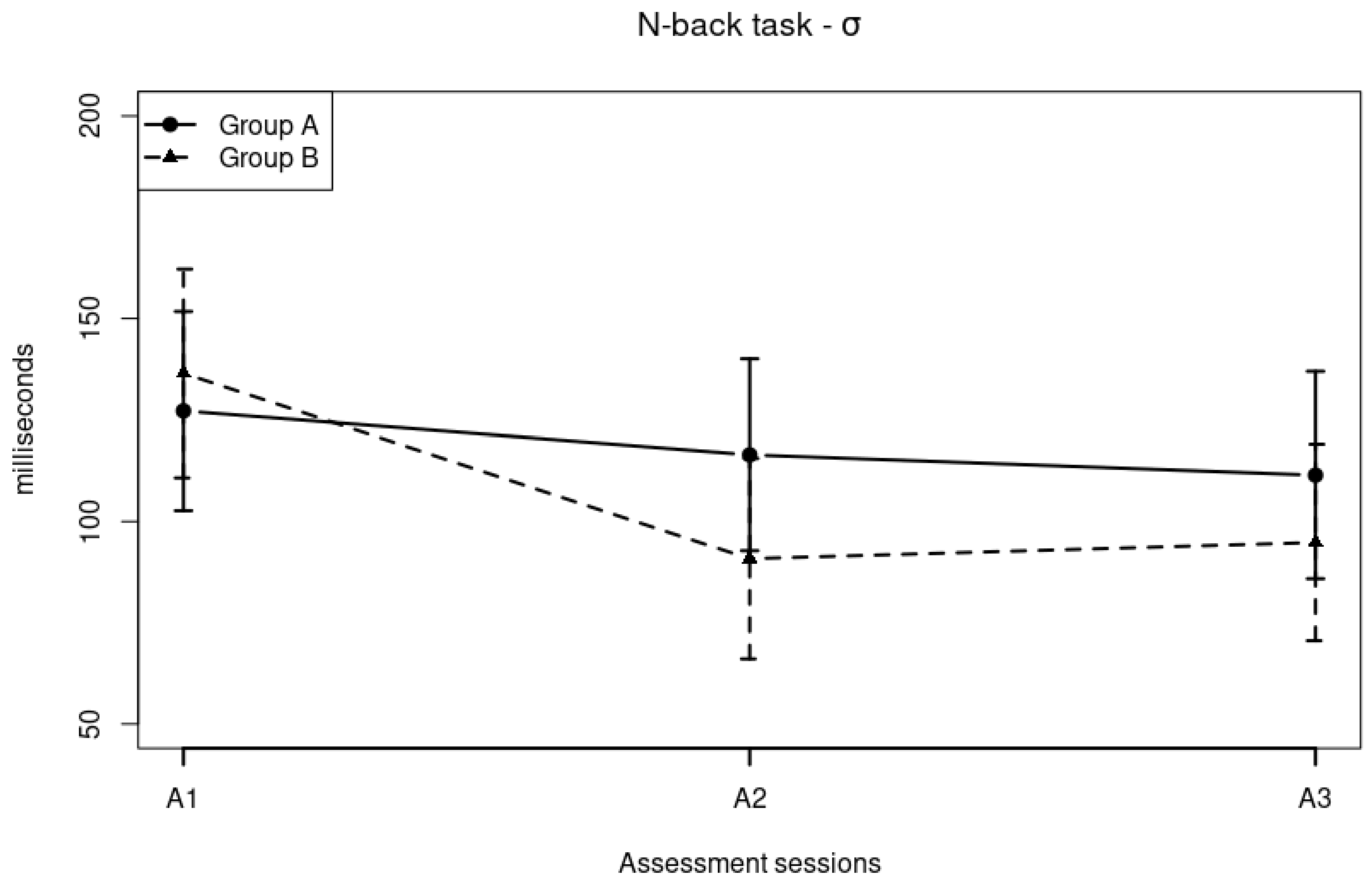

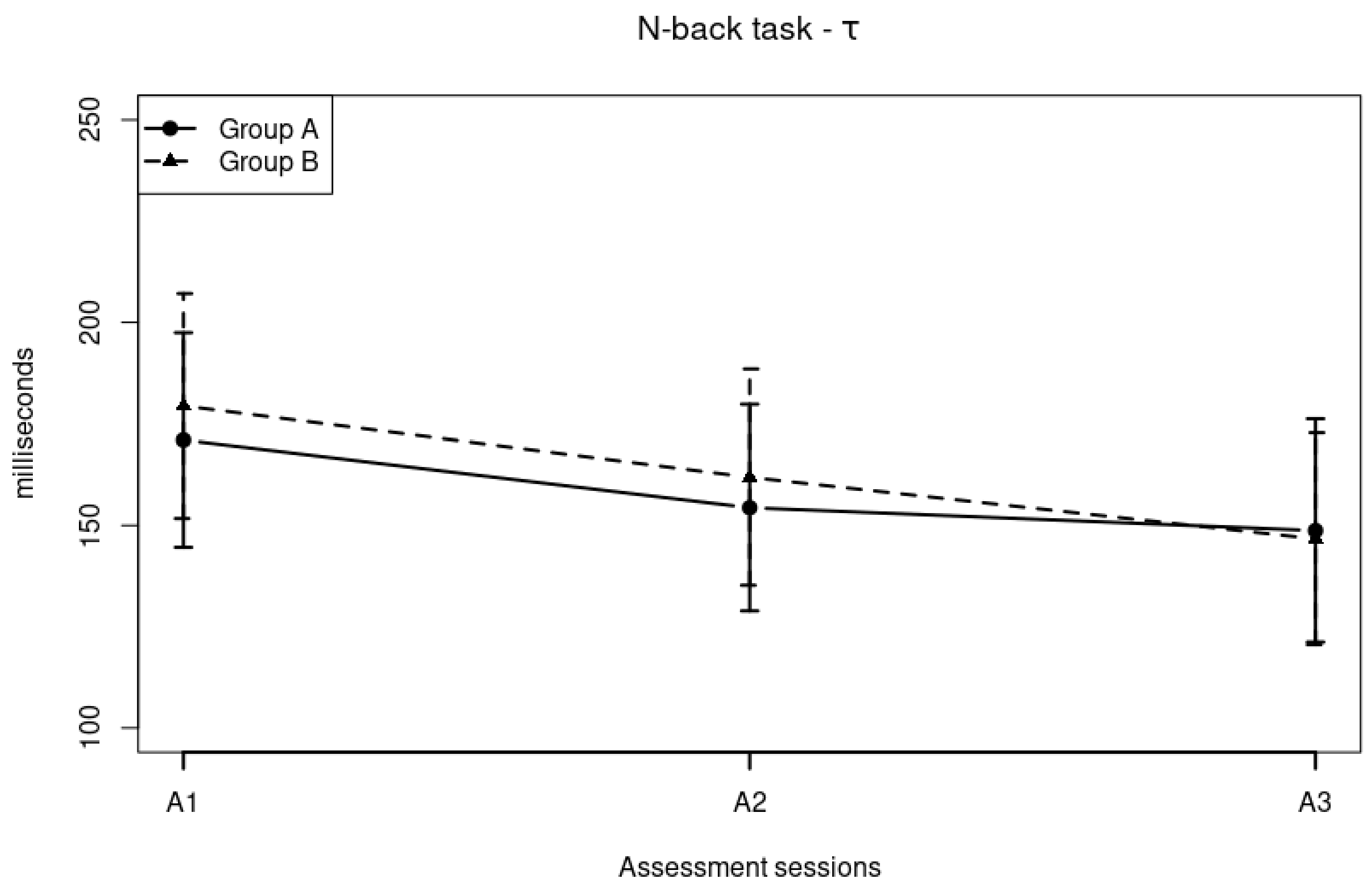
N-back task - τ
Group A
Group B
milliseconds
250
200
150
100
A1
A2
A3
Assessment sessions

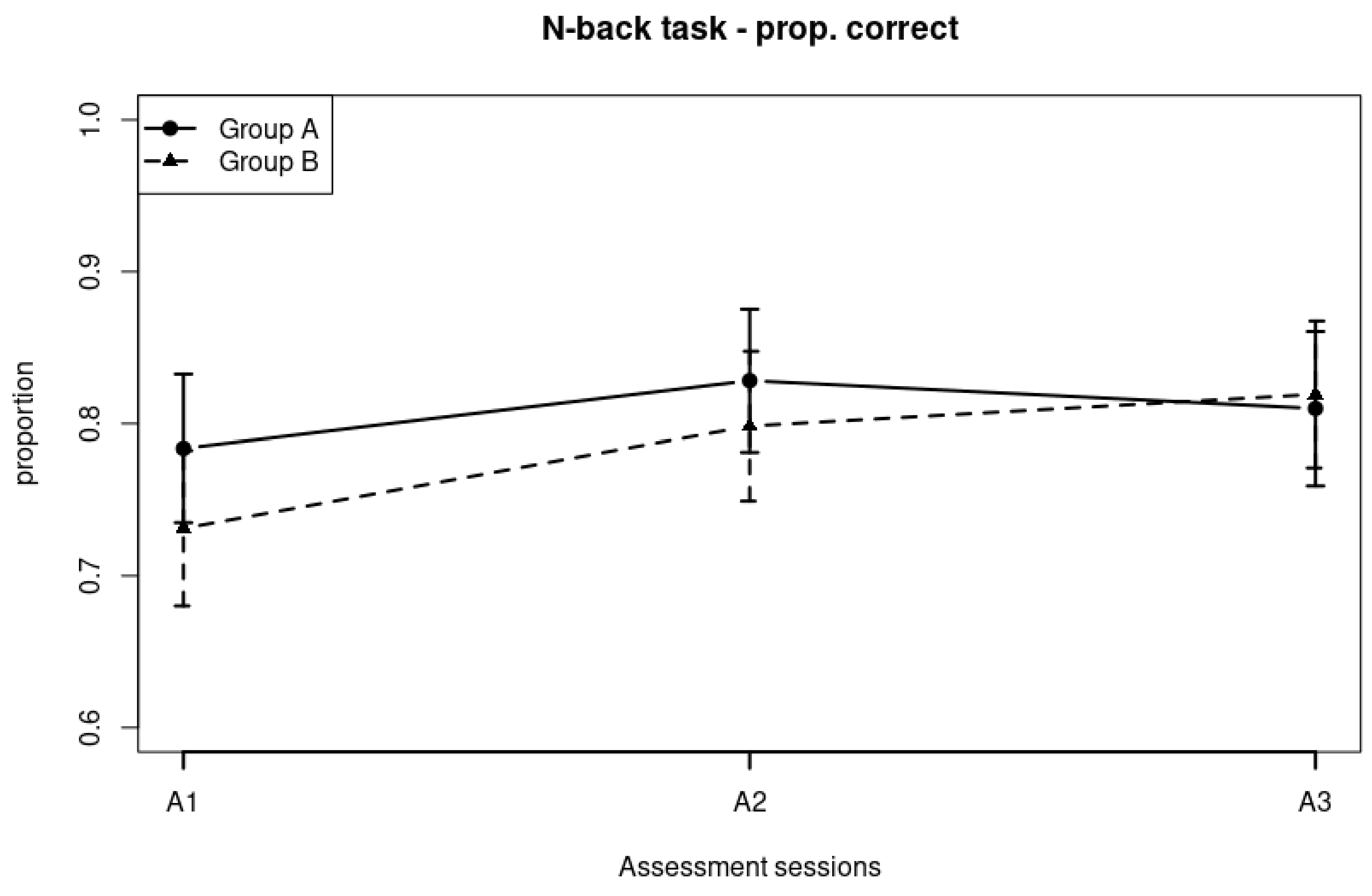
N-back task - prop. correct
Group A
Group B
proportion
1.0
0.9
0.8
0.7
0.6
A1
A2
A3
Assessment sessions

Stop-Signal task

	Go RT **μ**		Go RT **σ**		Go RT **τ**		SSRT	
	df	*F*	*df*	*F*	*df*	*F*	*df*	*F*
Session	2, 64.496	5.8137 ***	2, 66.803	7.8580 ***	2, 60.490	12.8062 ***	2, 55.436	33.710 *
Group	1, 38.791	20.5501 ***	1, 39.495	7.0759 *	1, 30.024	6.2112 *	1, 28.389	0.0216
Session* Group	2, 64.496	0.7594	2, 66.803	1.0272	2, 60.490	0.1884	2, 55.436	2.5278

*$p < 0.05$ **$p < 0.01$ ***$p < 0.001$

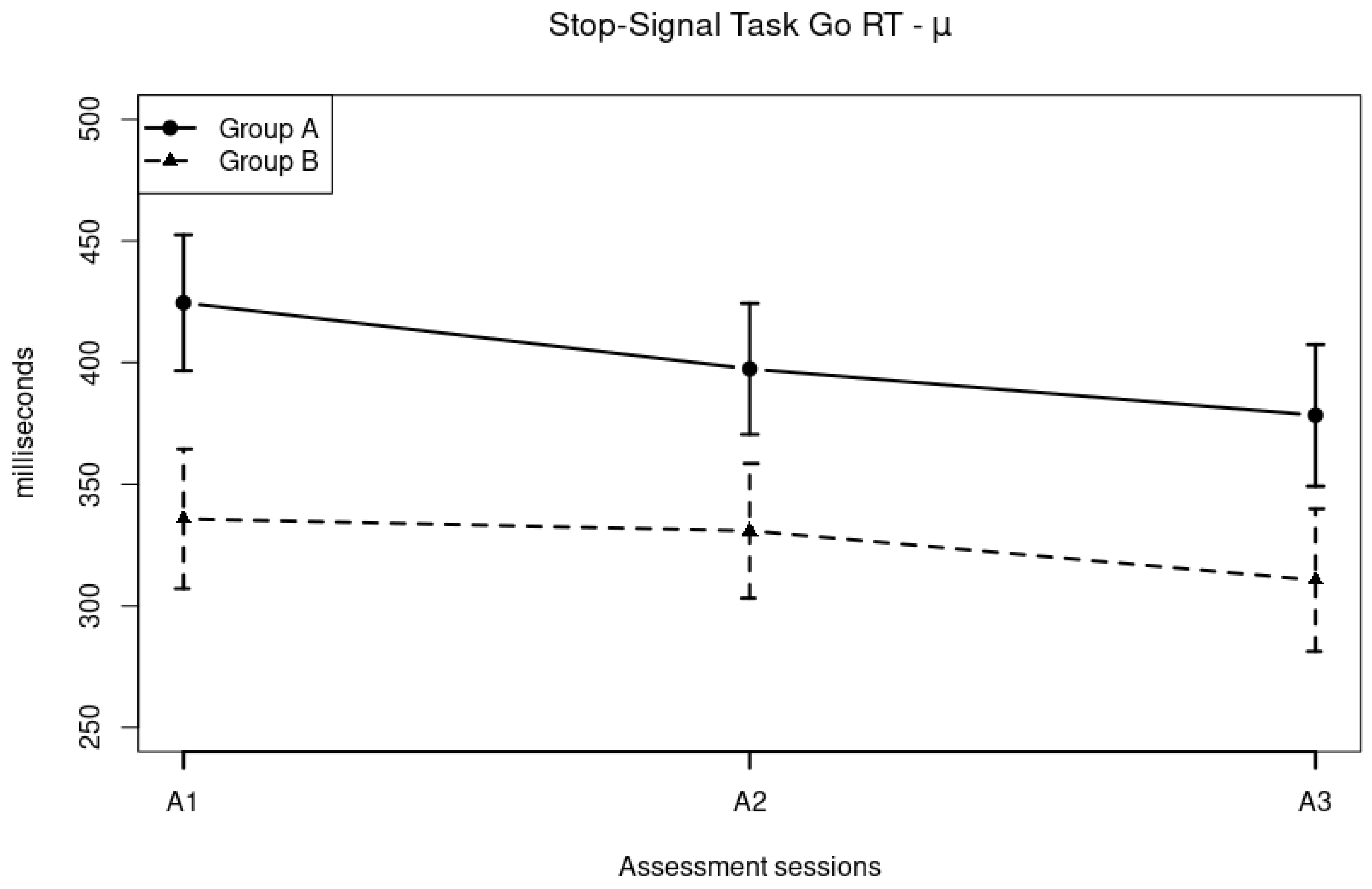

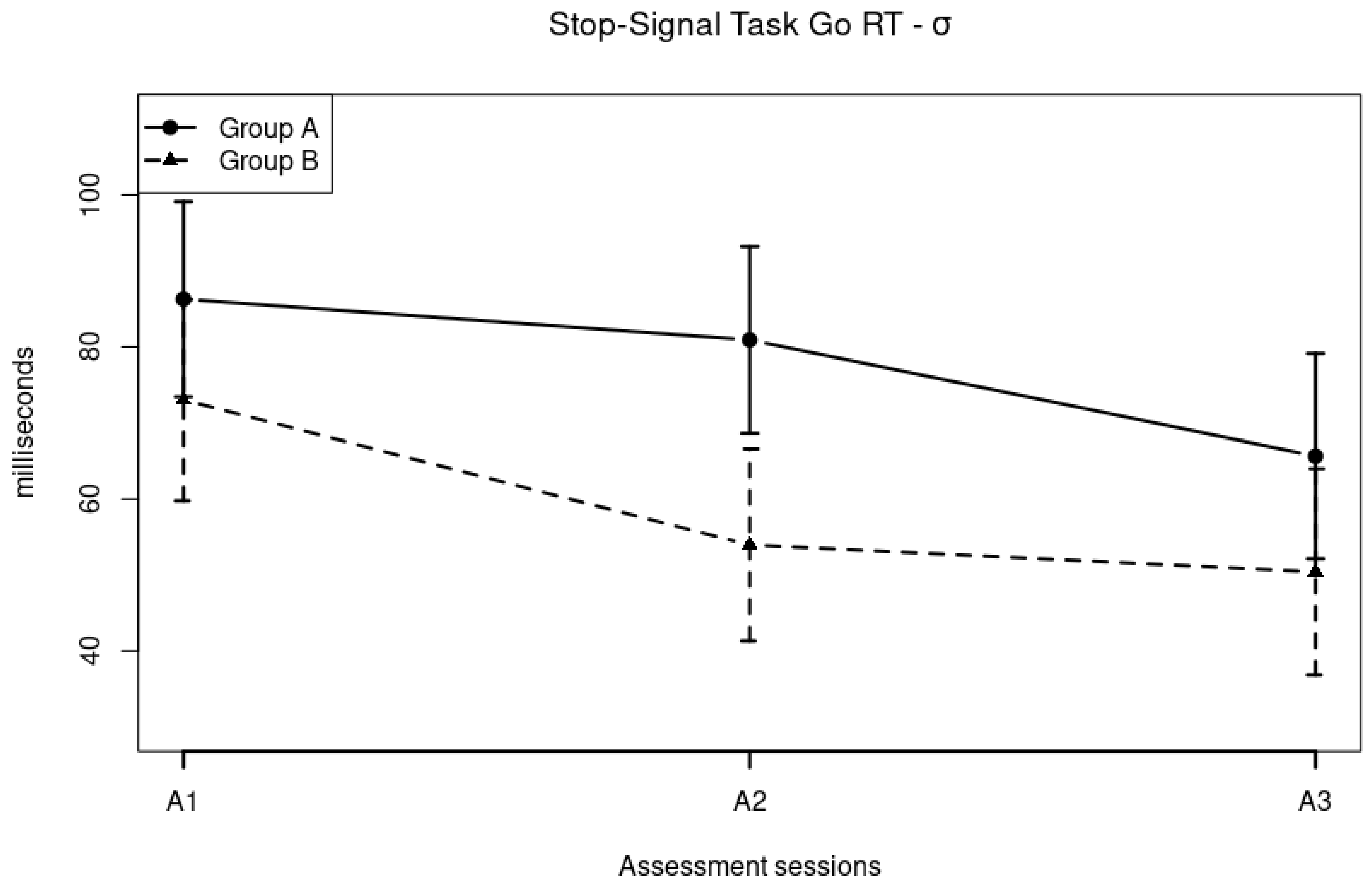

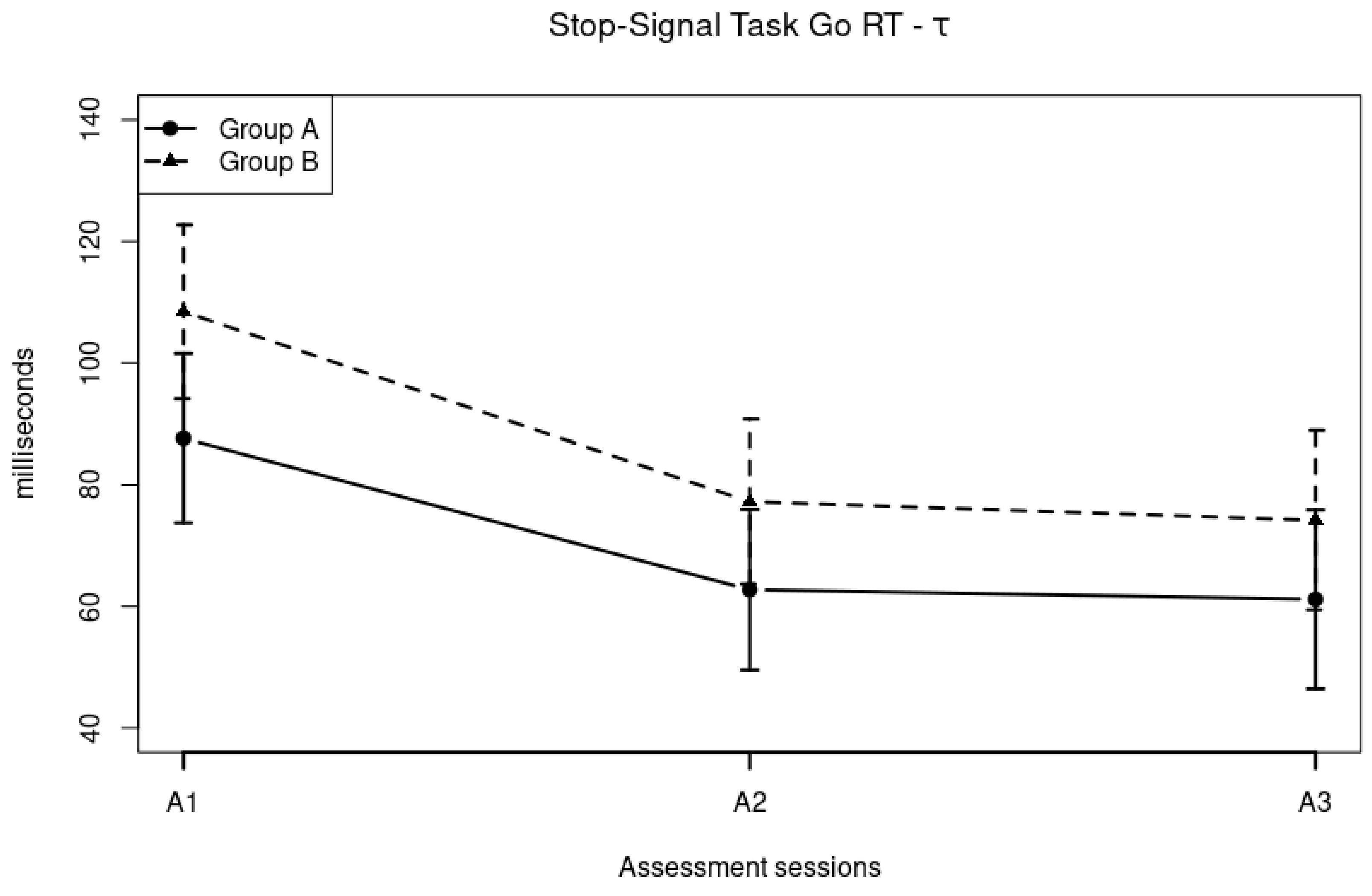
Stop-Signal Task Go RT - τ
Group A
Group B
milliseconds
140
120
100
80
60
40
A1
A2
A3
Assessment sessions

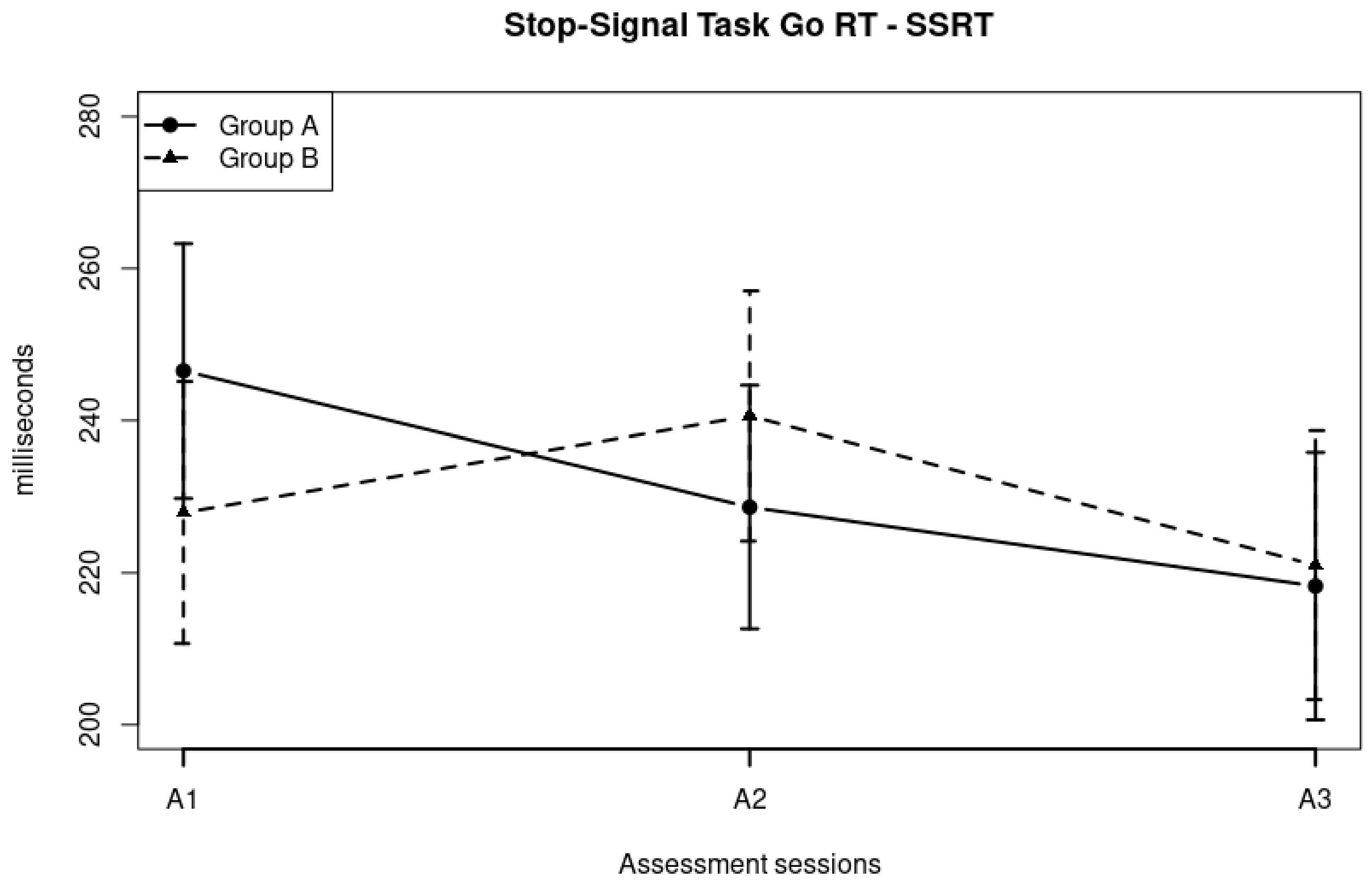
Stop-Signal Task Go RT - SSRT
Group A
Group B
milliseconds
280
260
240
220
200
A1
A2
A3
Assessment sessions

ANT Task

	RT **μ**		RT **σ**		RT **τ**		prop. correct	
	df	F	df	F	df	F	df	F
Session	2, 294.497	4.9744 **	2, 294.080	0.3649	2, 295.200	0.6383	2, 294.799	10.3960 ***
Group	1, 37.267	0.8580	1, 36.106	0.0041	1, 36.221	0.0078	1, 39.152	3.3395
Target	2, 289.198	21.5396 ***	2, 287.946	0.8071	2, 288.149	0.6444	2, 291.107	258.5536 ***
Session * Group	2, 294.497	1.0648	2, 294.080	0.6253	2, 295.200	1.2586	2, 294.799	1.5902
Session * Target	2, 289.198	0.2653	4, 287.946	0.1573	4, 288.149	0.3788	4, 291.107	5.0692 ***
Group * Target	2, 289.198	0.0622	2, 287.946	0.2568	2, 288.149	0.2055	2, 291.107	7.0791 ***
Session * Group * Target	4, 289.198	0.9478	4, 287.946	0.3217	4, 288.149	0.7813	4, 291.107	0.4749

*$p < 0.05$ **$p < 0.01$ ***$p < 0.001$

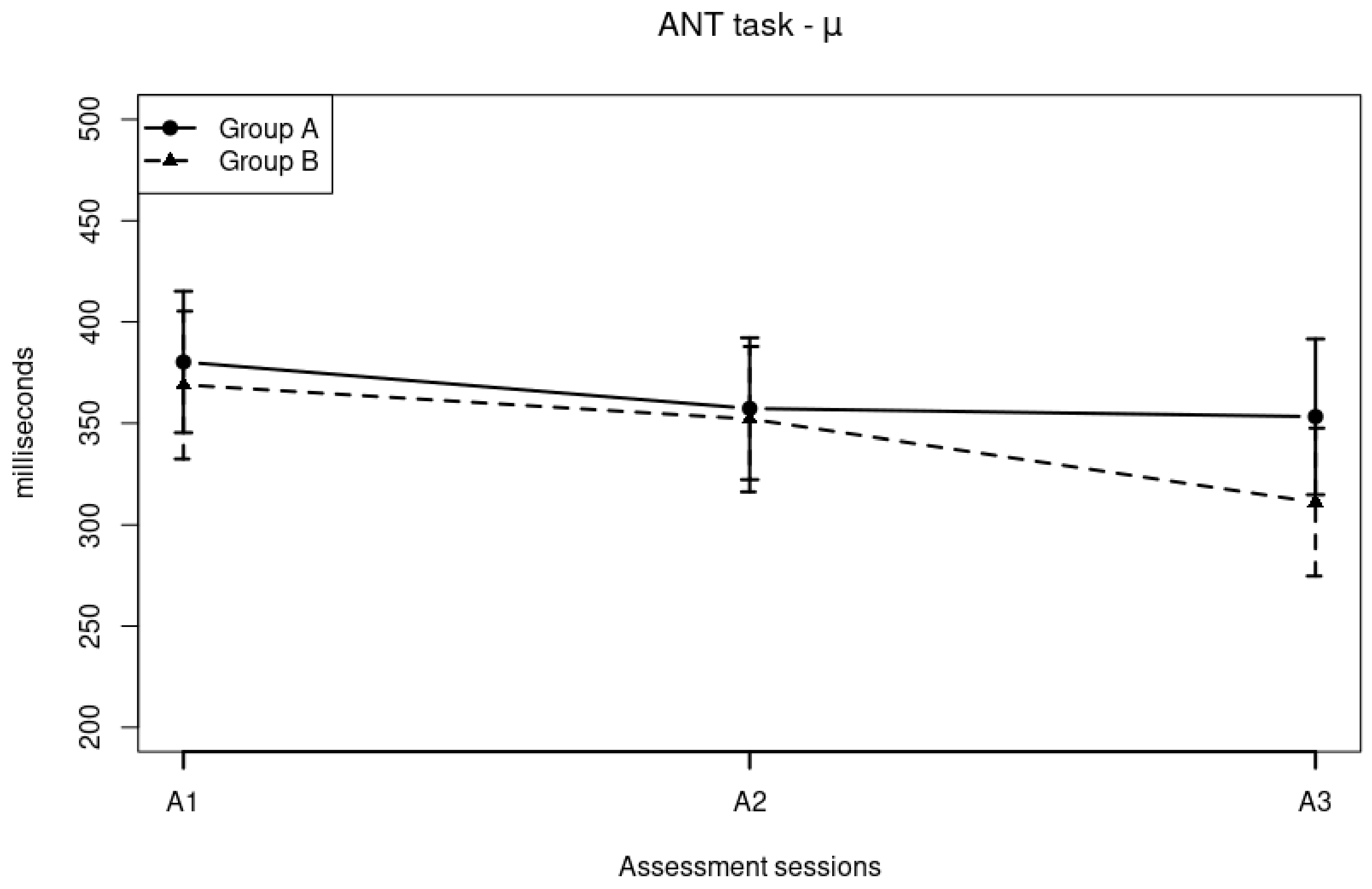

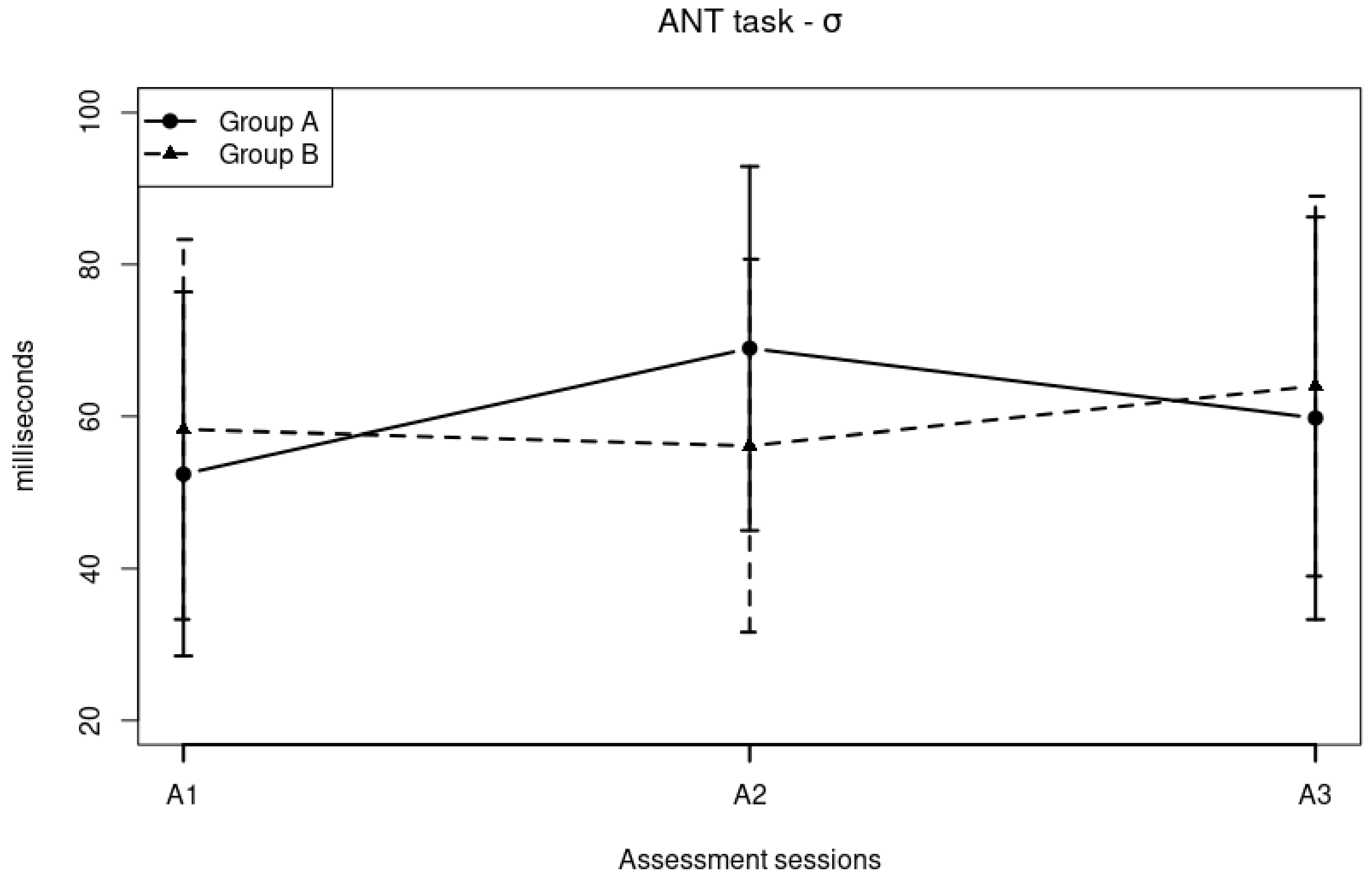

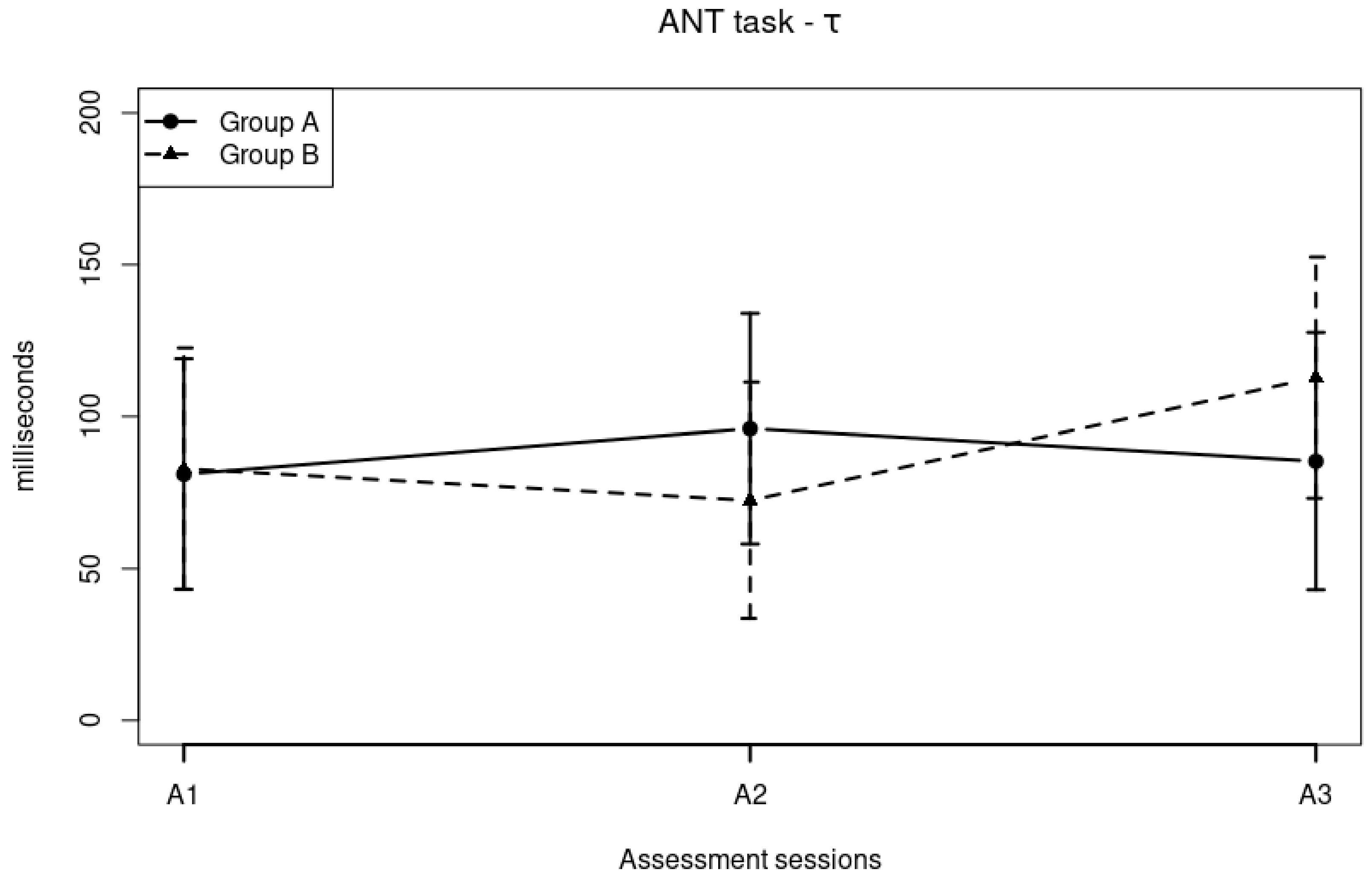
ANT task - τ
Group A
Group B
milliseconds
0
50
100
150
200
A1
A2
A3
Assessment sessions

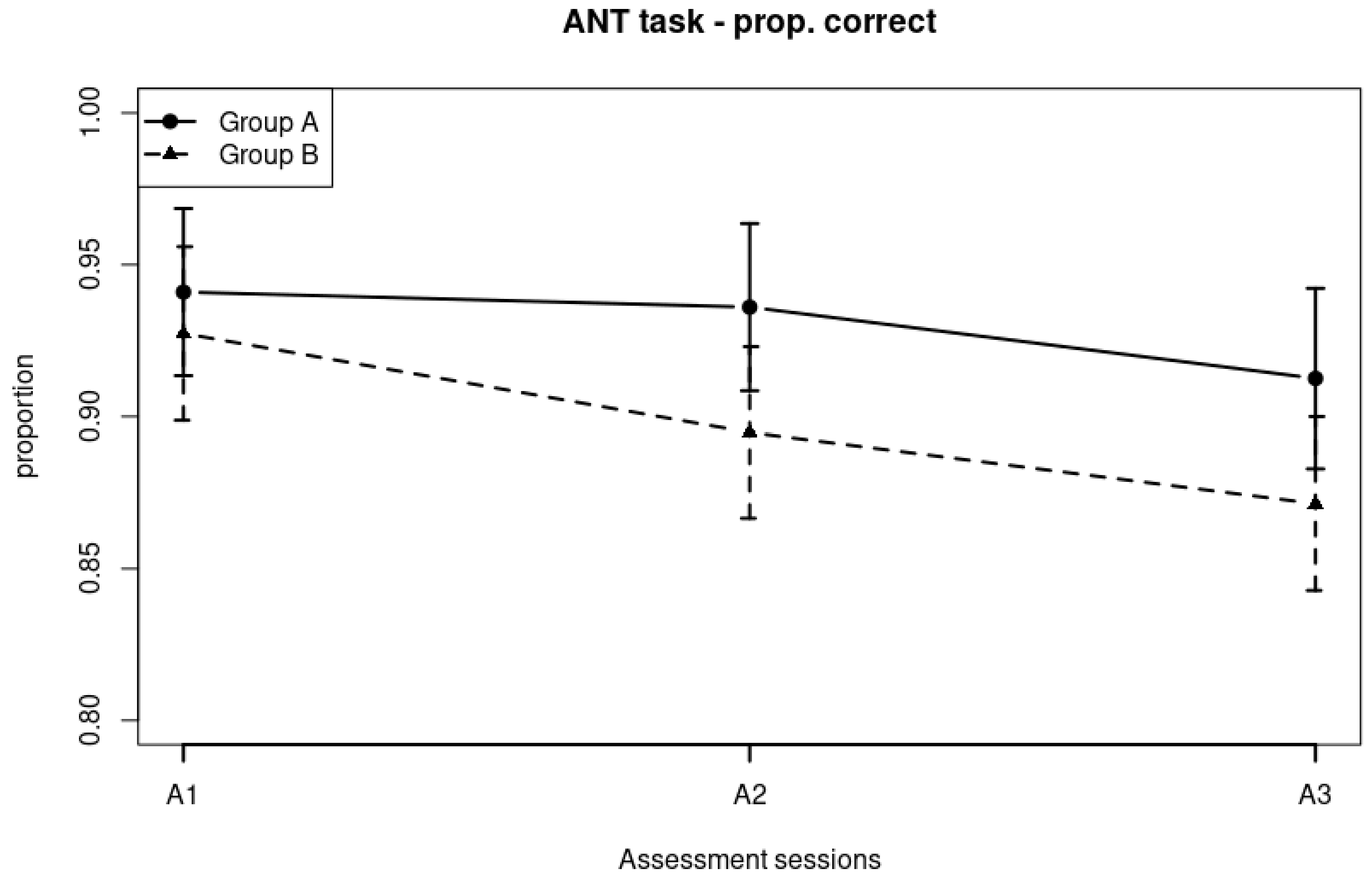
ANT task - prop. correct
Group A
Group B
proportion
0.80
0.85
0.90
0.95
1.00
A1
A2
A3
Assessment sessions

Switching task

	RT **μ**		RT **σ**		RT **τ**		prop. correct	
	df	F	df	F	df	F	df	F
Session	2, 370.62	66.0476 ***	2, 373.43	45.9709 ***	2, 376.81	37.5947 ***	2, 375.43	11.6701 ***
Group	1 , 34.14	1.2220	1, 34.21	0.8484	1, 35.02	6.1285 *	1, 35.51	3.8943
Switch	1, 368.10	331.4338 ***	1, 368.20	33.1599 ***	1, 369.13	12.0706 ***	1, 369.53	21.2978 ***
Session * Group	2, 370.62	12.0847 ***	2, 373.43	8.2721 ***	2, 376.81	15.2115 ***	2, 375.43	34.5260 ***
Session * Switch	2, 368.10	0.6908	2, 368.20	1.9718	2, 369.13	0.9582	2, 369.53	3.4716
Group * Switch	1, 368.10	1.2405	1, 368.20	5.1042 *	1, 369.13	0.1743	1, 369.53	0.3480
Session * Group * Switch	2, 368.10	0.4775	2, 368.20	2.5510	2, 369.13	0.3317	2, 369.53	0.5079

$^*p < 0.05$ $^{**}p < 0.01$ $^{***}p < 0.001$

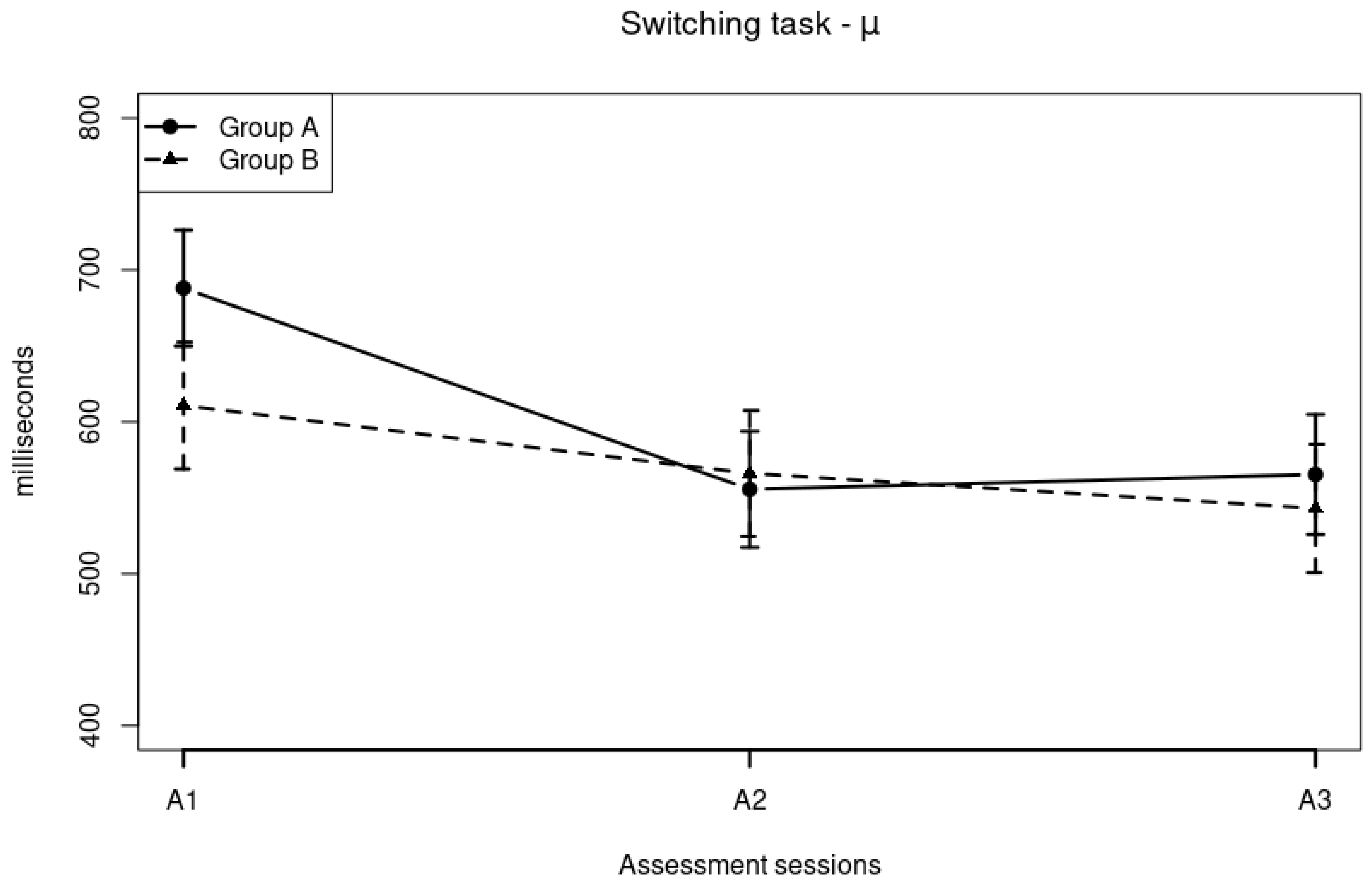

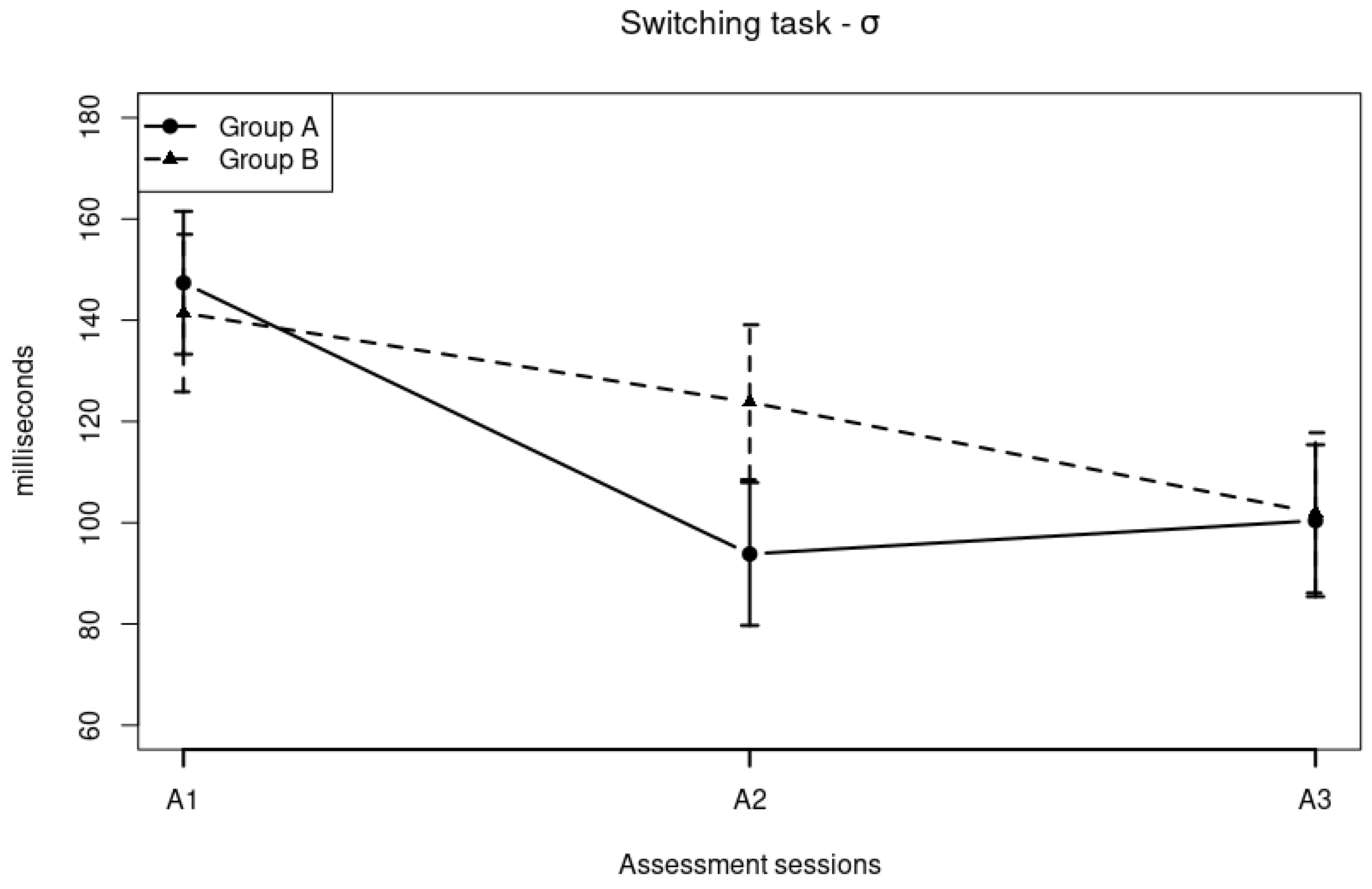

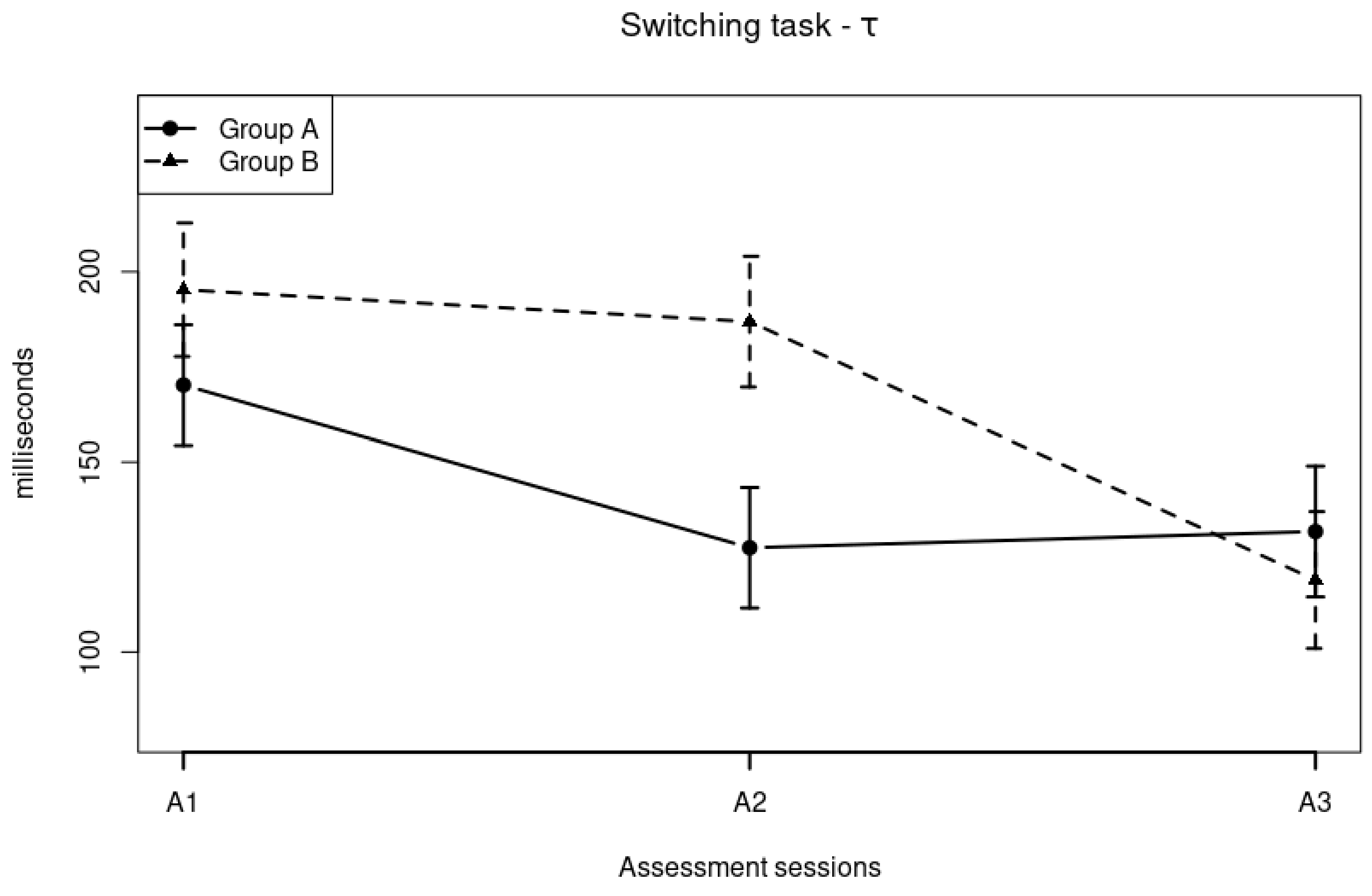
Switching task - τ
Group A
Group B
milliseconds
200
150
100
A1
A2
A3
Assessment sessions

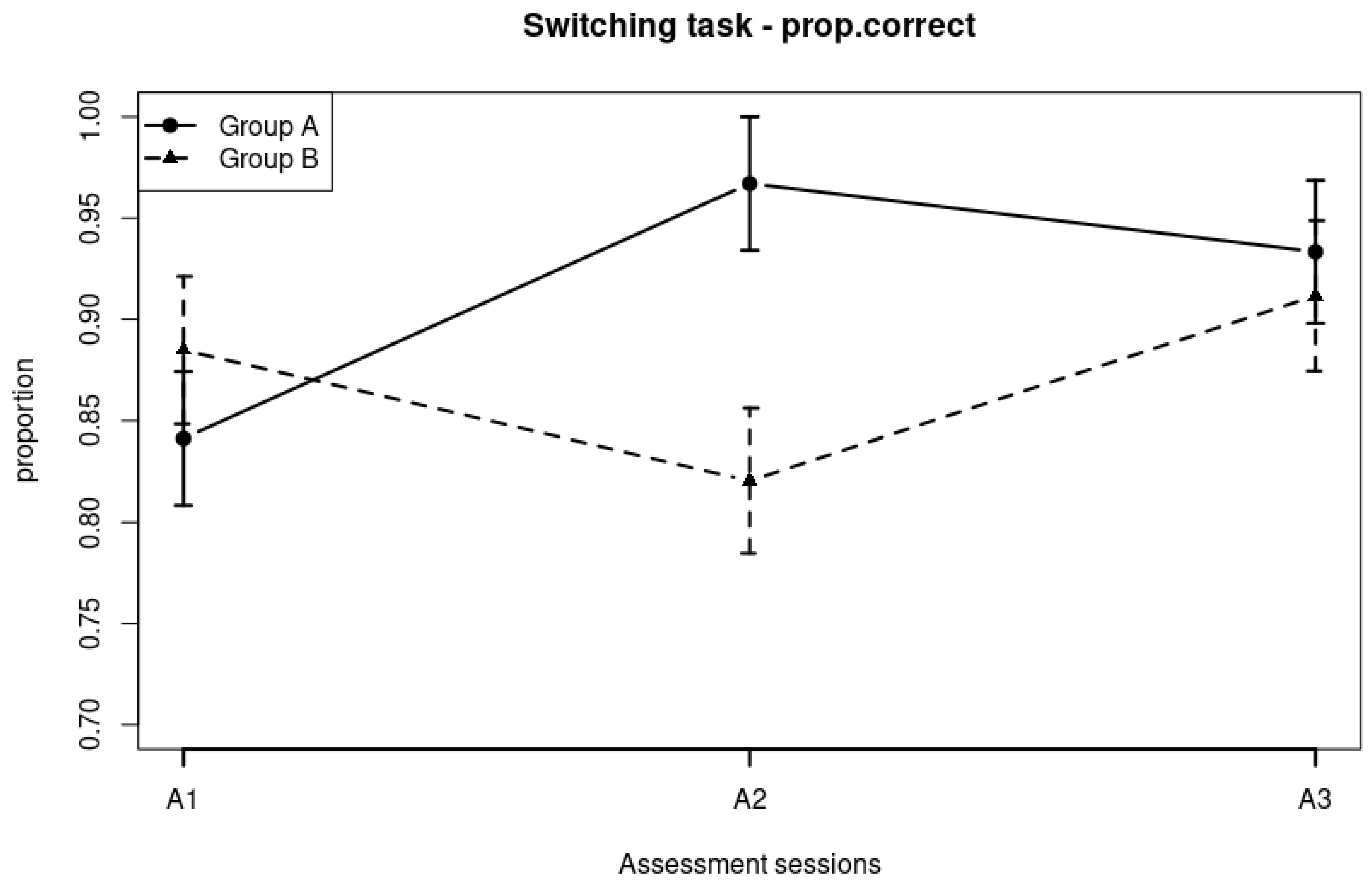
Switching task - prop.correct
Group A
Group B
proportion
1.00
0.95
0.90
0.85
0.80
0.75
0.70
A1
A2
A3
Assessment sessions

Psychomotor vigilance task

	RT **μ**		RT **σ**		RT **τ**		prop. correct	
	df	*F*	*df*	*F*	*df*	*F*	*df*	*F*
Session	2, 301.550	0.3517	2, 314.857	3.1880 *	2, 317.95	2.6845	2, 65.606	3.7857 *
Group	1, 38.939	1.2413	1, 27.305	4.6336 *	1, 33.49	8.1684 **	1, 33.945	1.1632
Session* Group	2, 301.550	0.8615	2, 314.857	1.1405	2, 317.95	1.2703	2, 65.606	1.9489

*$p < 0.05$ **$p < 0.01$ ***$p < 0.001$

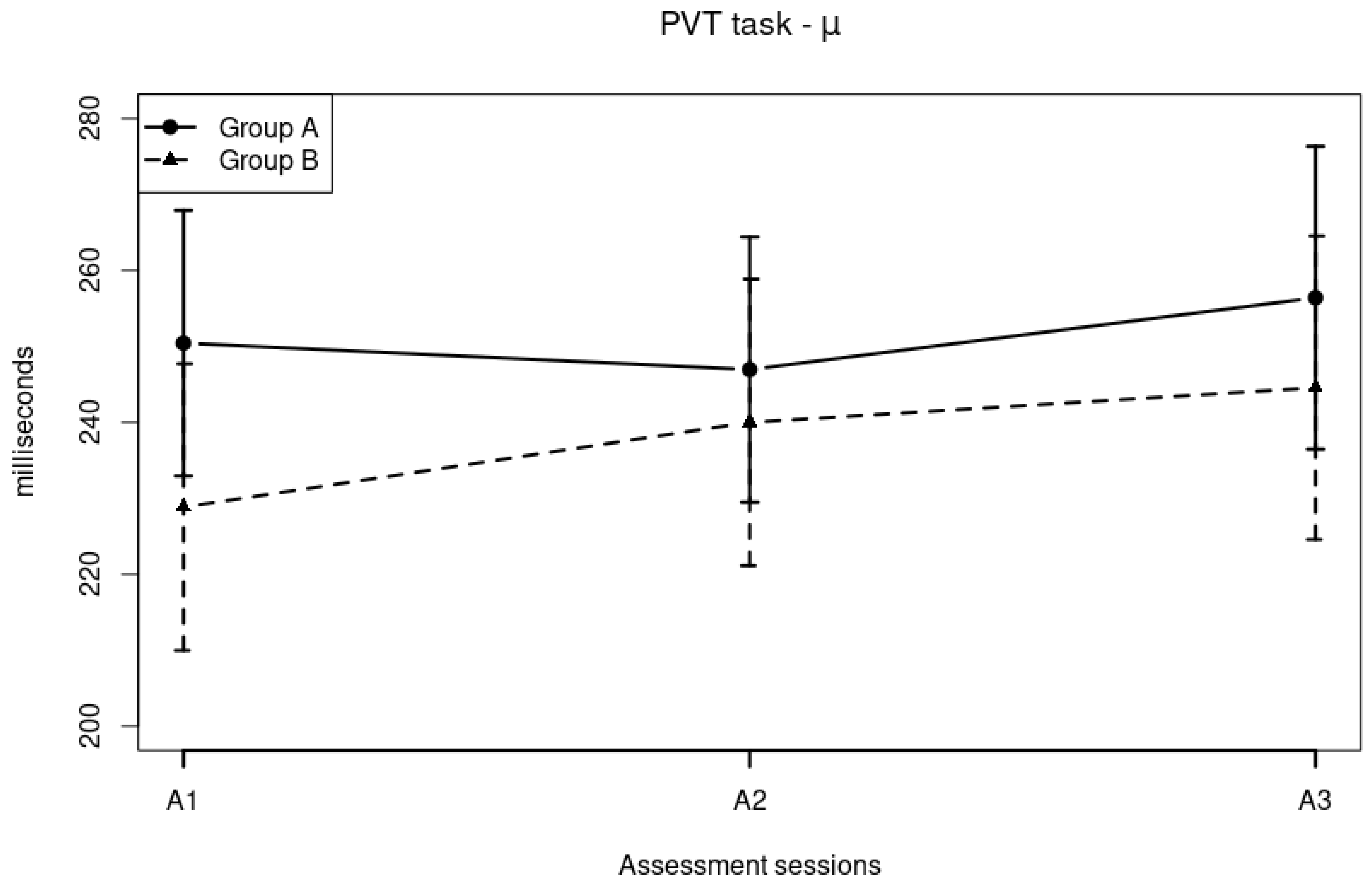

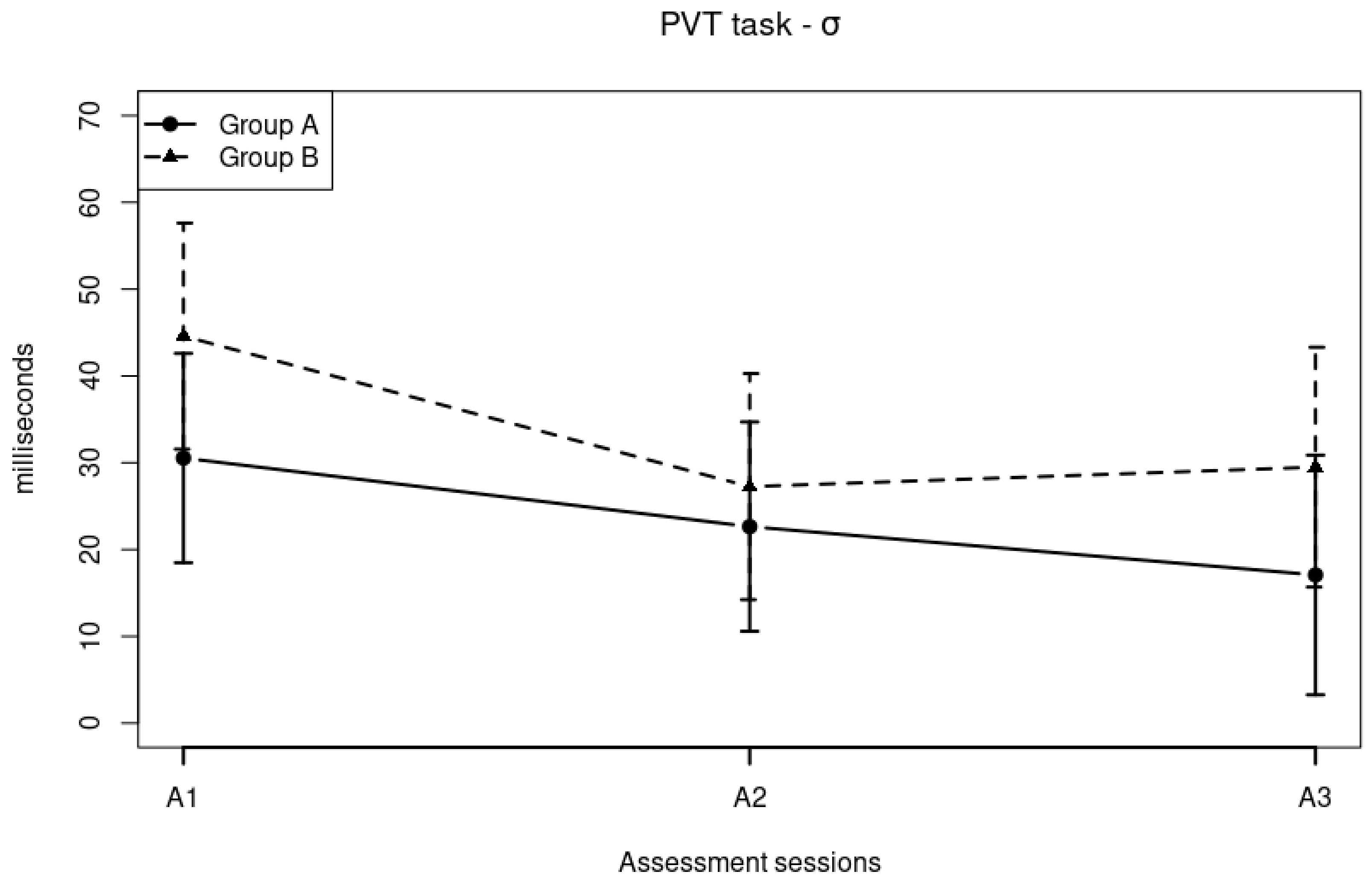

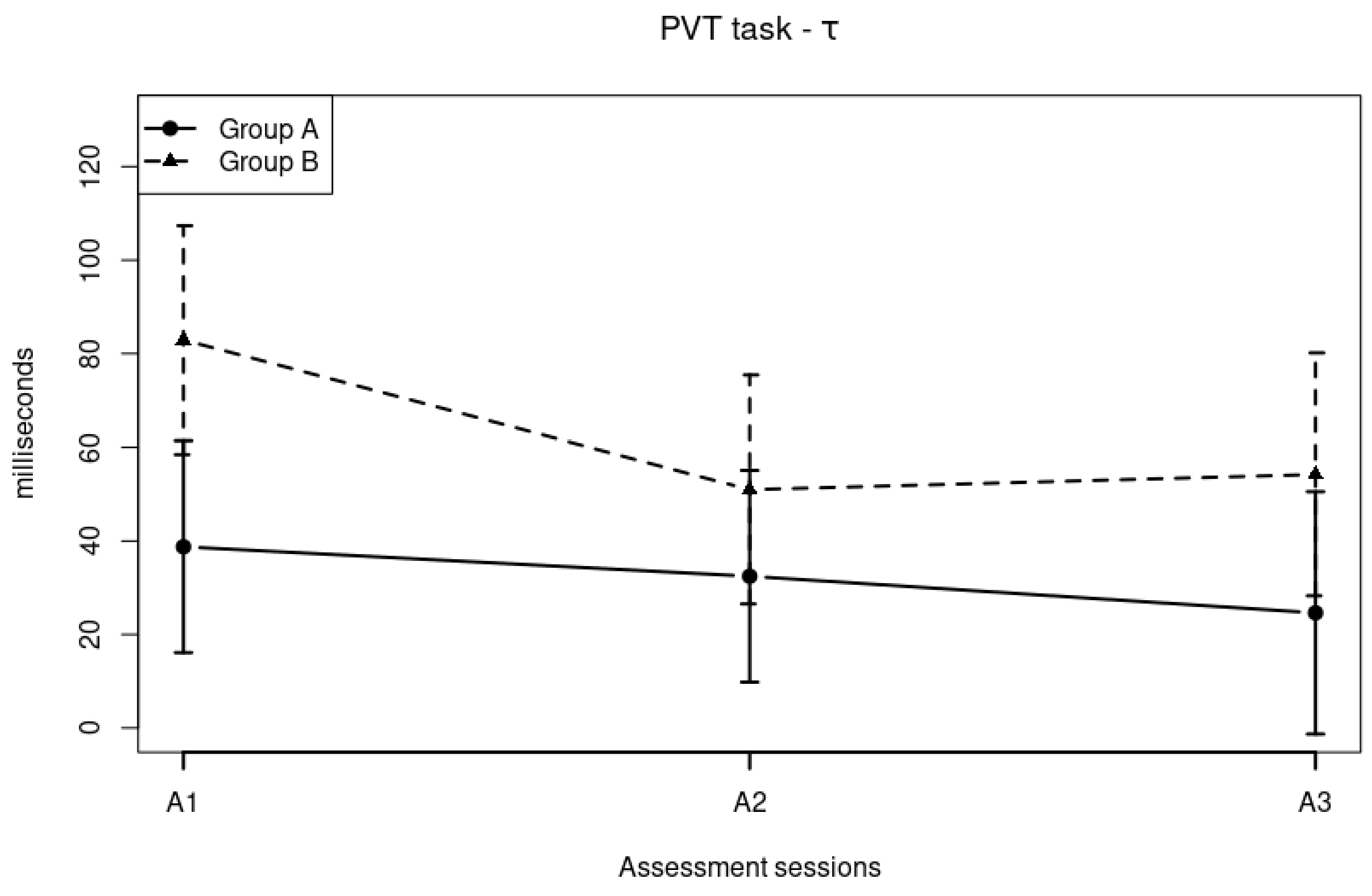
PVT task - τ
Group A
Group B
milliseconds
0
20
40
60
80
100
120
A1
A2
A3
Assessment sessions

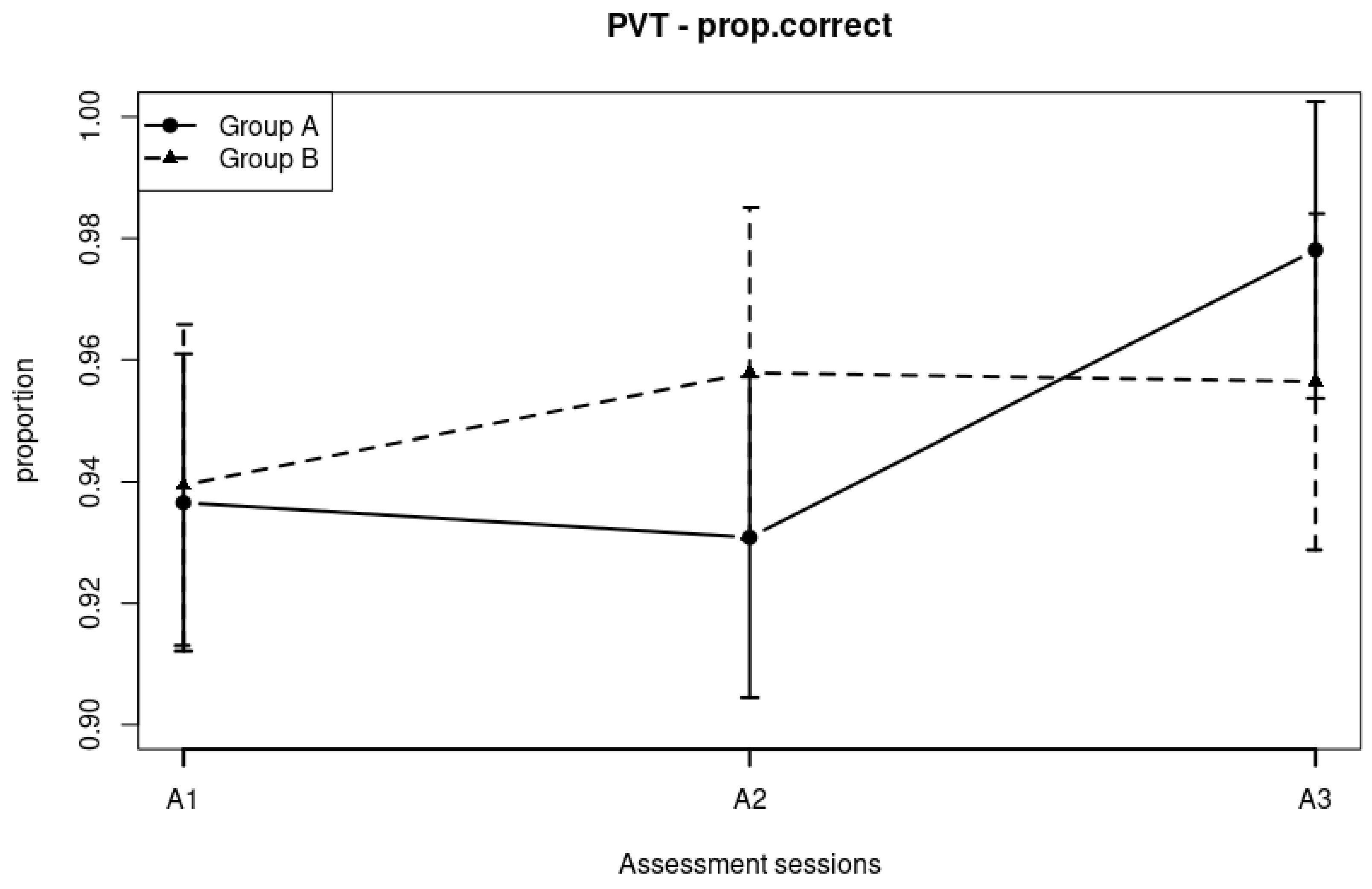
PVT - prop.correct
Group A
Group B
proportion
0.90
0.92
0.94
0.96
0.98
1.00
A1
A2
A3
Assessment sessions

Mental rotation task

	RT **μ**		RT **σ**		RT **τ**		prop. correct	
	df	*F*	*df*	*F*	*df*	*F*	*df*	*F*
Session	2, 176.842	2.3181	2, 173.606	5.8988 **	2, 175.51	4.1685 *	2, 64.949	15.6035 ***
Group	1, 36.684	0.8826	1, 29.839	3.7076	1, 29.51	8.5135 **	1, 34.862	2.9488
Session* Group	2, 176.842	2.6557	2, 173.606	2.1072	2, 175.51	2.2231	2, 64.949	9.5638 ***

$*p < 0.05$ $**p < 0.01$ $***p < 0.001$

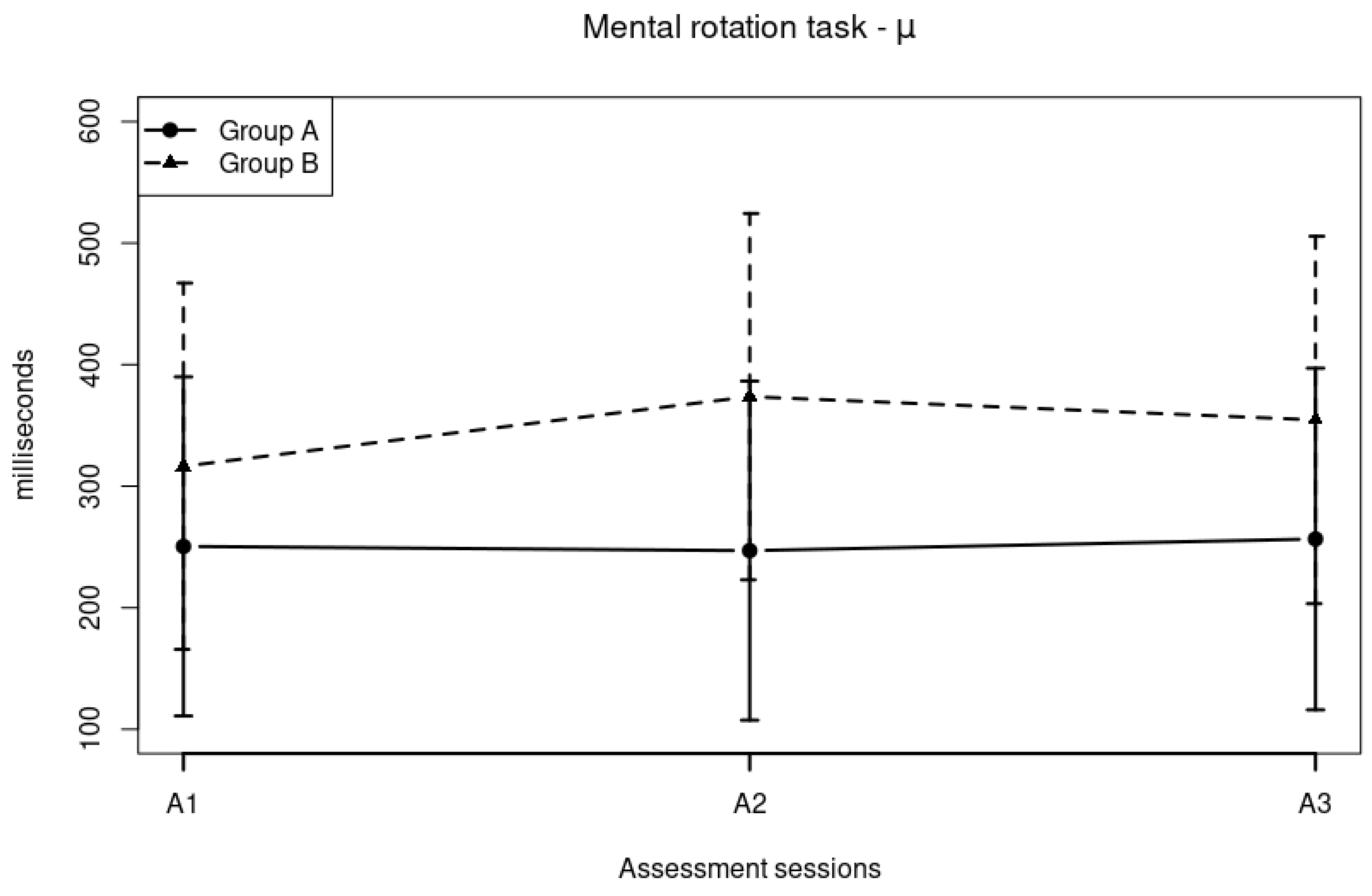

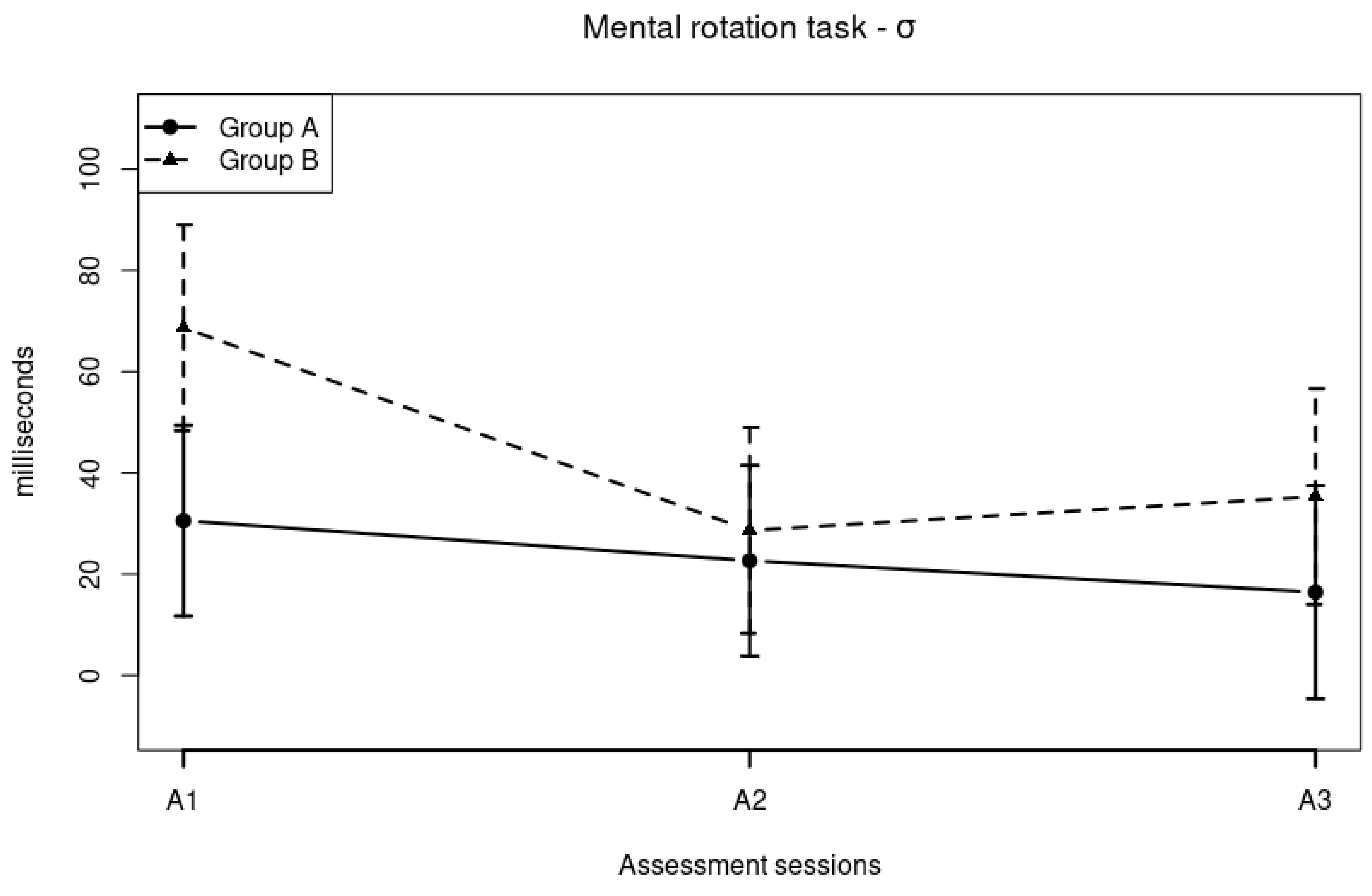

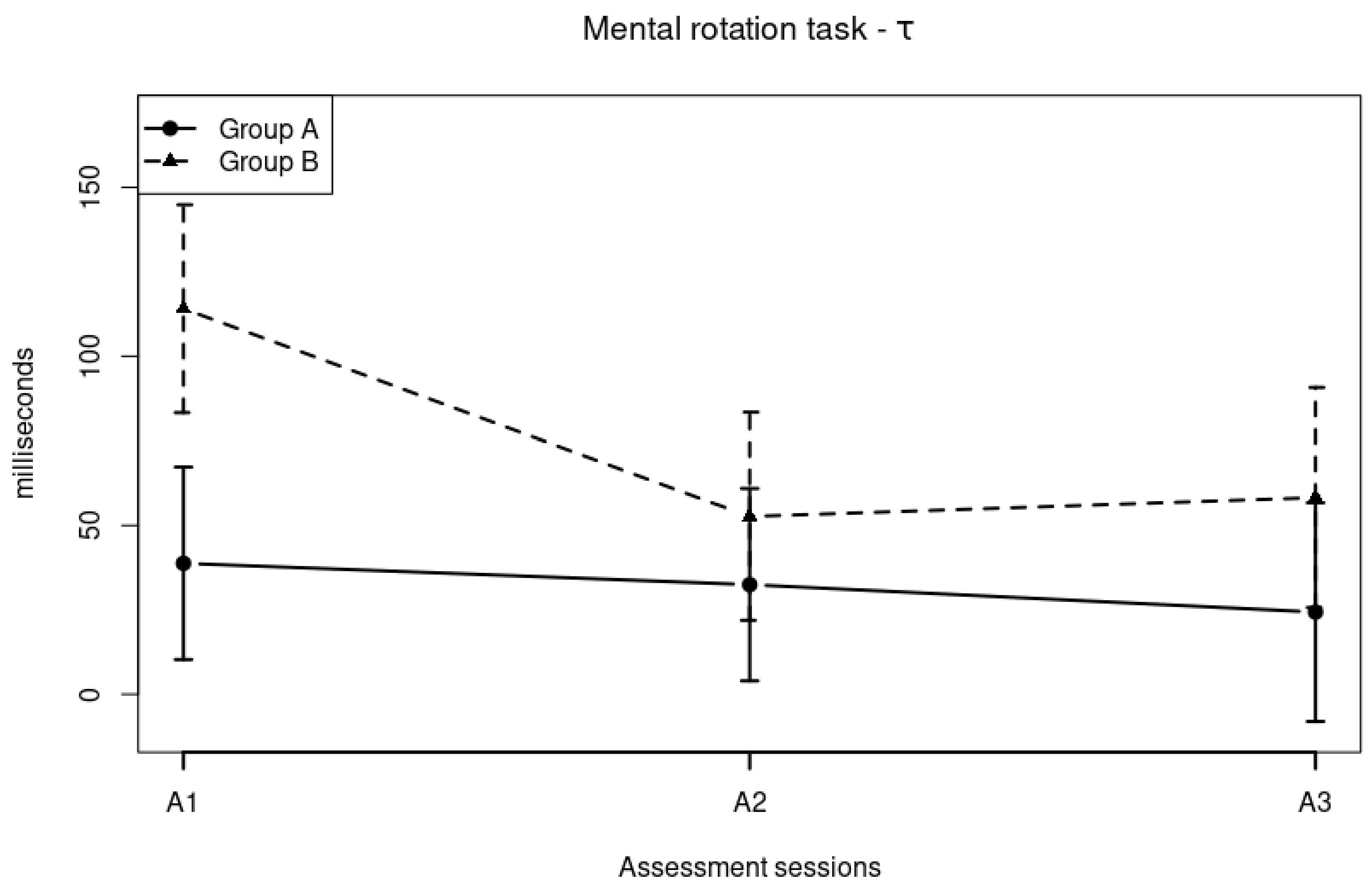
Mental rotation task - τ
Group A
Group B
milliseconds
0
50
100
150
A1
A2
A3
Assessment sessions

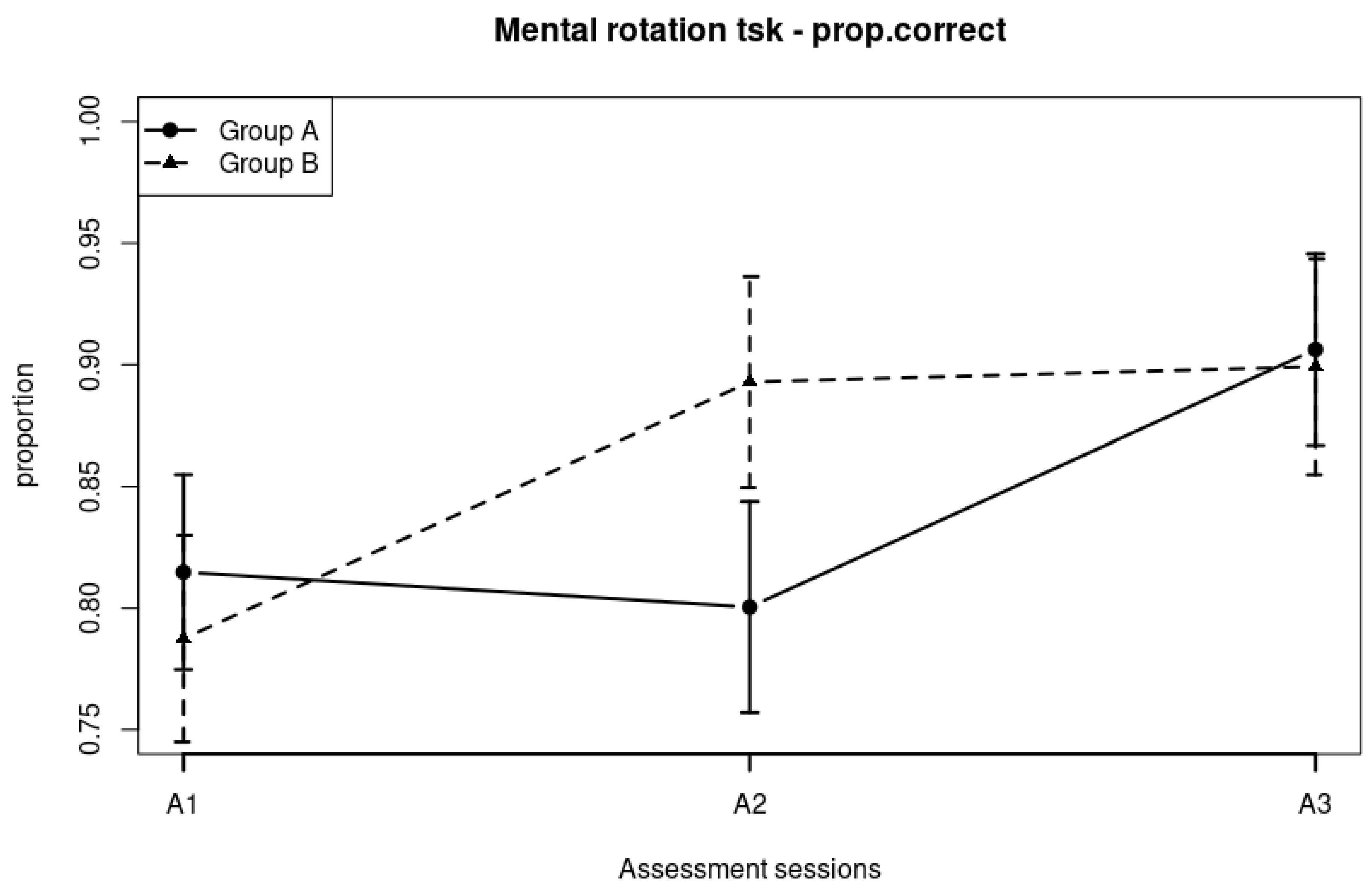
Mental rotation tsk - prop.correct
Group A
Group B
proportion
0.75
0.80
0.85
0.90
0.95
1.00
A1
A2
A3
Assessment sessions
