# Supplementary material for: Alpha Neurofeedback Training in Elite Soccer Players Trained in Groups
Source: Appl Psychophysiol Biofeedback. 2024 Aug 10;49(4):589–602. doi: 10.1007/s10484-024-09654-1 (PMC11588833; doi:10.1007/s10484-024-09654-1)

Workload (NASA-TLX)

	Session		Group		Session * Group	
	df	*F*	*df*	*F*	*df*	*F*
Mental Demand	2, 73.053	0.4609	1, 39.787	0.5268	2, 73.053	0.4552
Physical Demand	2, 71.559	2.4707	1, 38.524	1.4259	2, 71.559	1.6325
Temporal Demand	2, 72.562	0.6899	1, 39.293	0.0023	2, 72.562	0.4053
Performance	2, 75.191	0.3407	1, 40.346	1.5761	2, 75.191	1.2586
Effort	2, 74.016	4.0898 *	1 ,40.288	3.3624	2, 74.016	0.0299
Frustration	2, 72.331	0.5586	1, 39.174	0.1406	2, 72.331	0.3071

*$p < 0.05$ **$p < 0.01$ ***$p < 0.001$

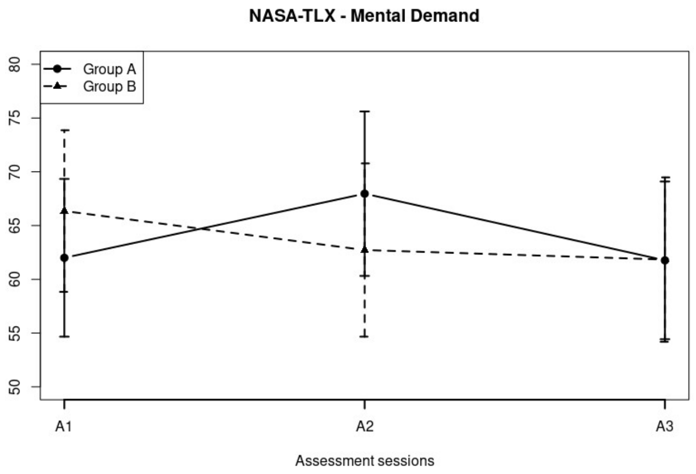

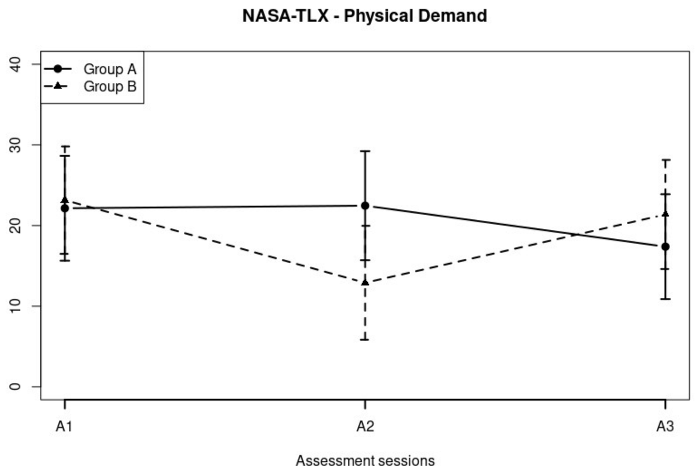

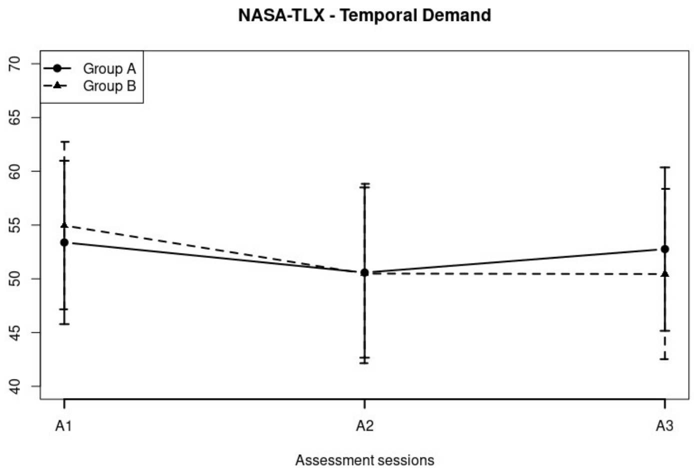

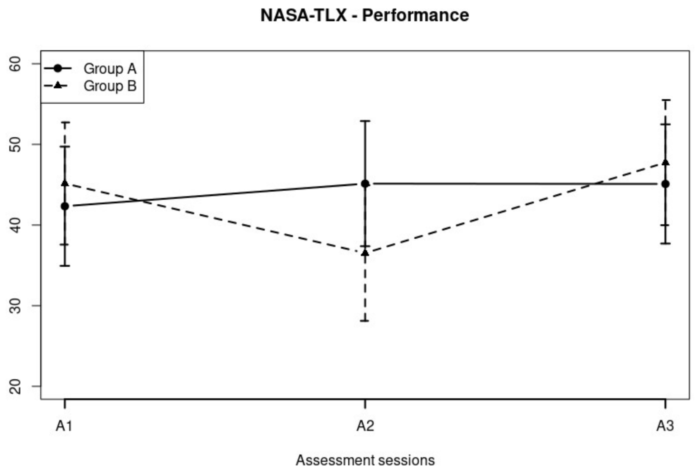

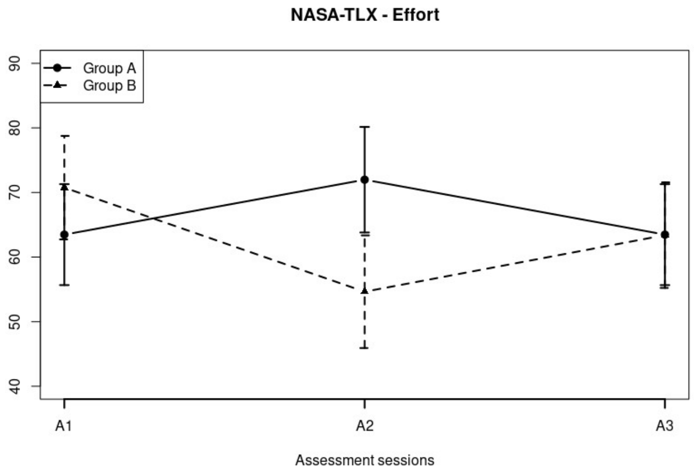

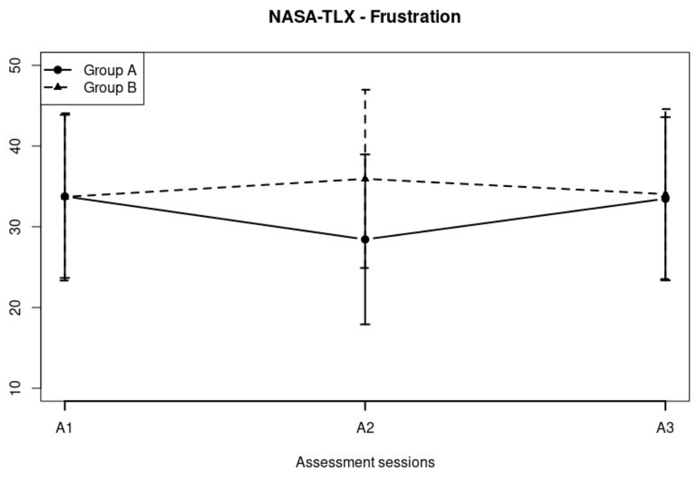

Mood (POMS)

	Session		Group		Session * Group	
	df	*F*	*df*	*F*	*df*	*F*
Tension/Anxiety	2, 73.286	0.6755	1, 39.683	0.7638	2, 73.286	0.3560
Anger/Hostility	2, 74.056	0.2580	1, 40.251	0.2284	2, 74.056	0.0992
Vigor/Activity	2, 72.425	2.7084	1, 38.525	0.2140	2, 72.425	1.3437
Fatigue/Inertia	2, 74.090	5.8872 **	1, 39.227	1.9509	2, 74.090	1.8551
Depression/Dejection	2, 73.008	0.2216	1, 39.283	0.1671	2, 73.008	1.2117

*$p < 0.05$ **$p < 0.01$ ***$p < 0.001$

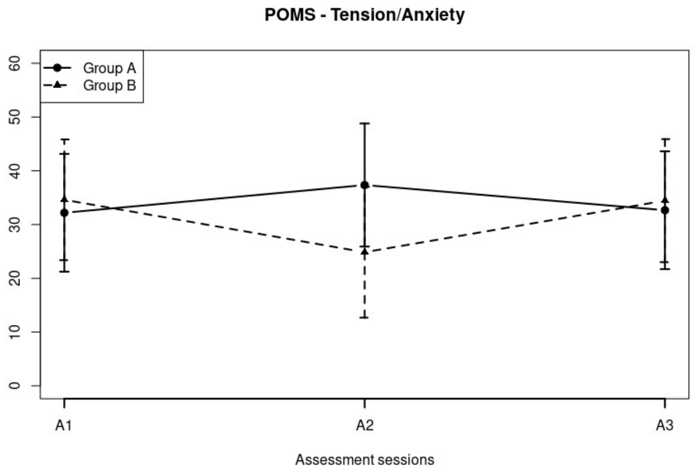

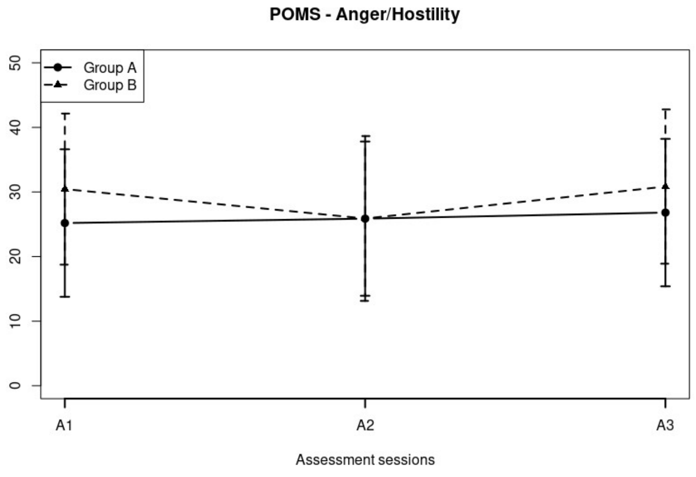

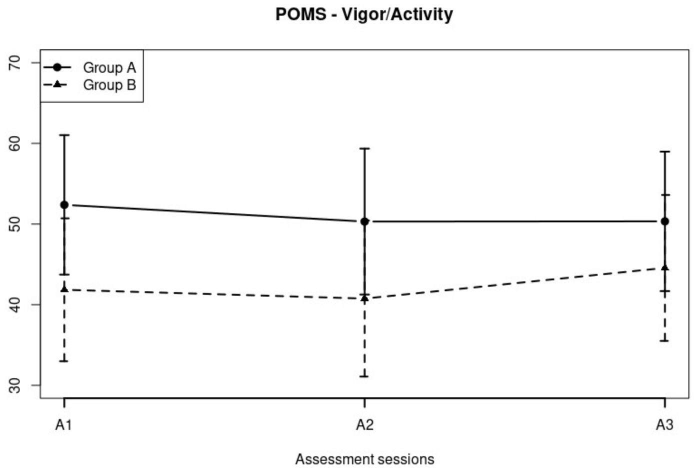

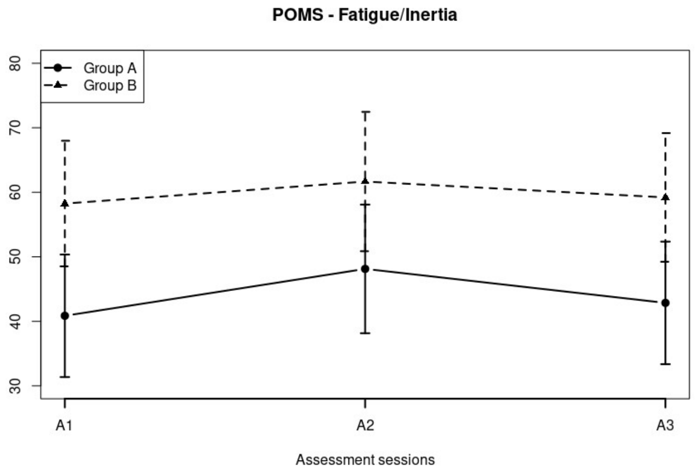

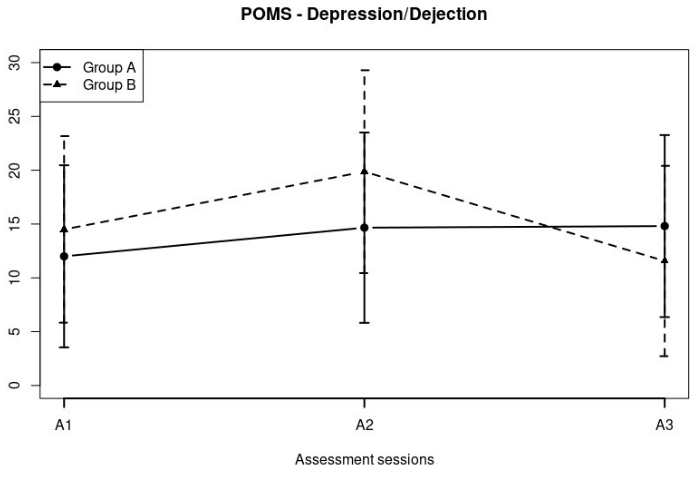

Stress (PSS-10)

	Session		Group		Session * Group	
	df	*F*	*df*	*F*	*df*	*F*
Perceived helplessness	2, 72.436	0.5195	1, 39.607	0.0050	2, 72.436	0.8018
Lack of self-efficacy	2, 72.510	1.0156	1, 39.200	0.4261	2, 72.510	0.5153

*$p < 0.05$ **$p < 0.01$ ***$p < 0.001$

PSS-10 - Perceived helplessness

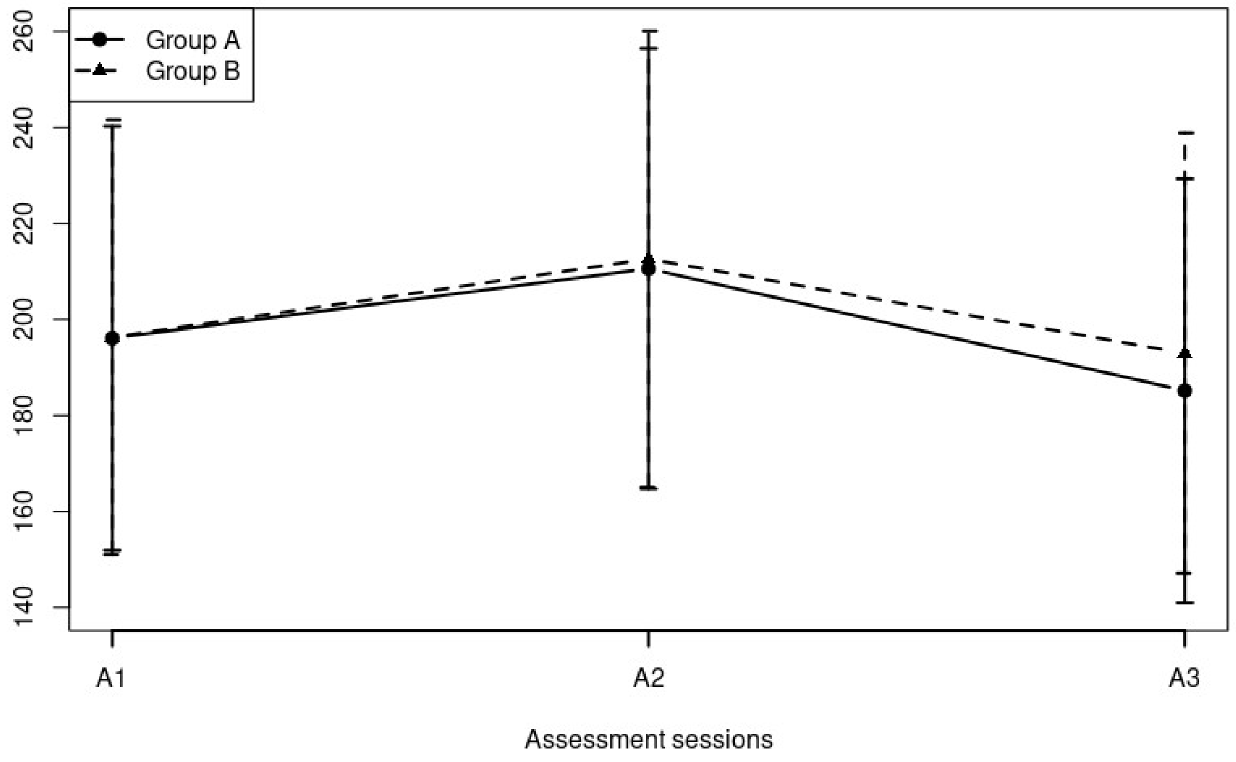

PSS-10 - Lack of self-efficacy

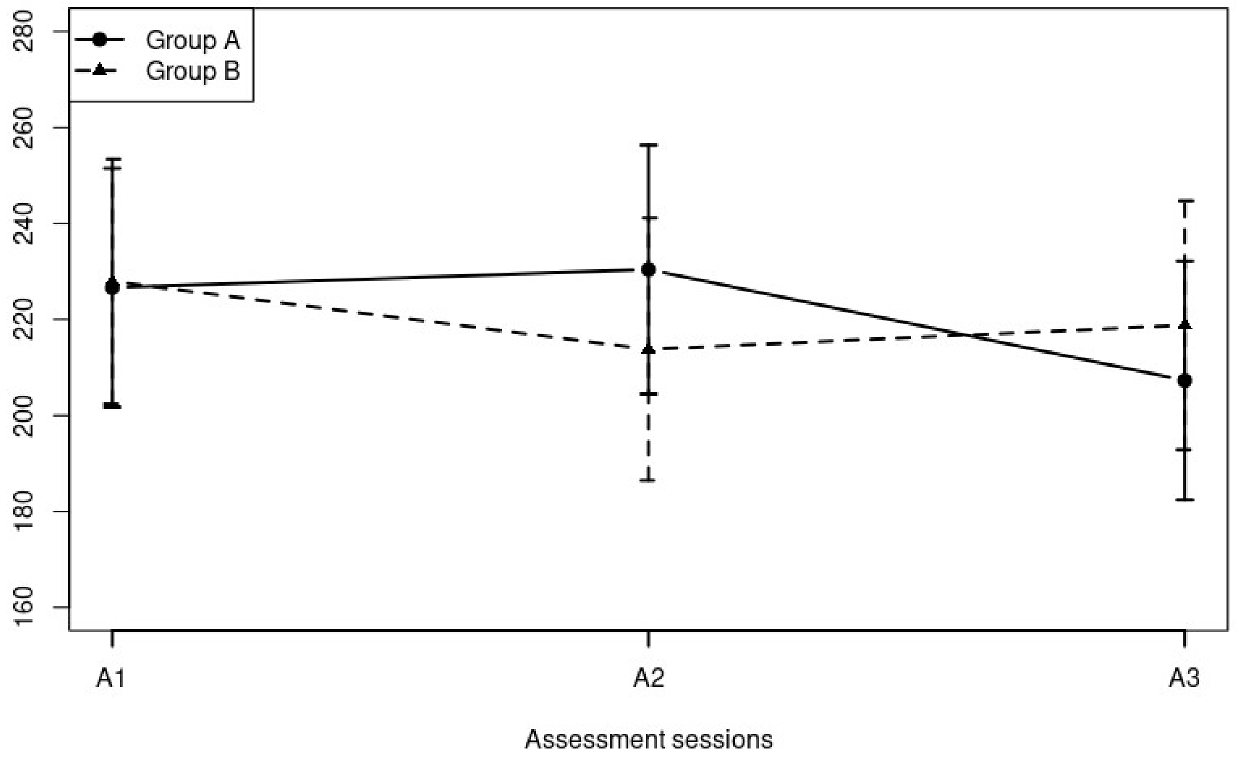

Sleep (ASSQ)

	Session		Group		Session * Group	
	df	*F*	*df*	*F*	*df*	*F*
Sleep duration	2, 72.733	1.1716	1, 39.617	0.0005	2, 72.733	4.7522 *
Time to fall asleep	2, 71.703	1.6051	1, 39.259	0.1931	2, 71.703	2.0861
Sleep satisfaction	2, 73.903	1.4633	1, 39.869	0.3940	2, 73.903	0.0705

$^{*}p < 0.05$ $^{**}p < 0.01$ $^{***}p < 0.001$

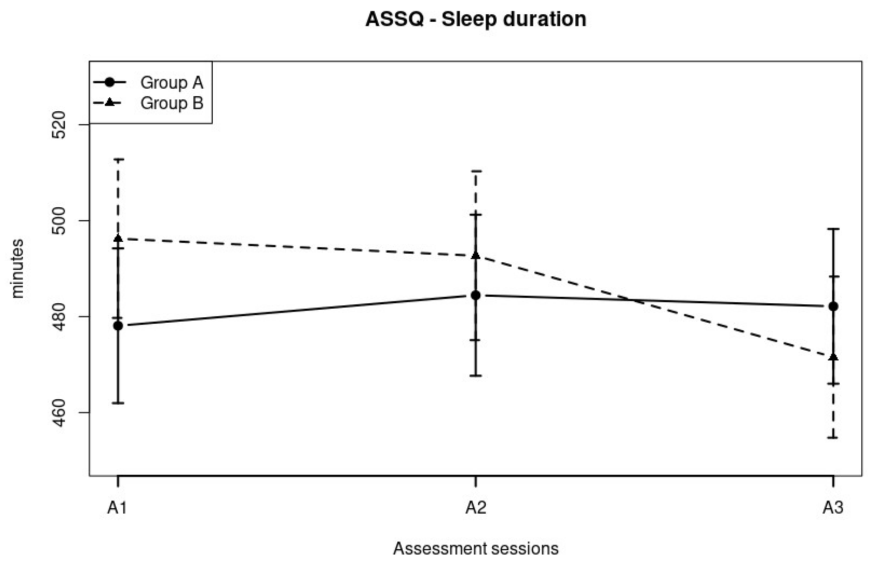

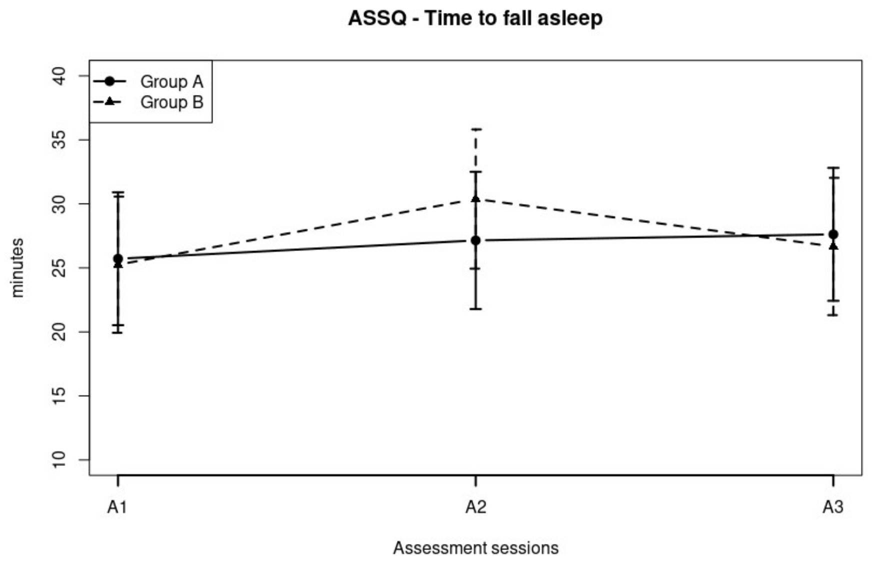

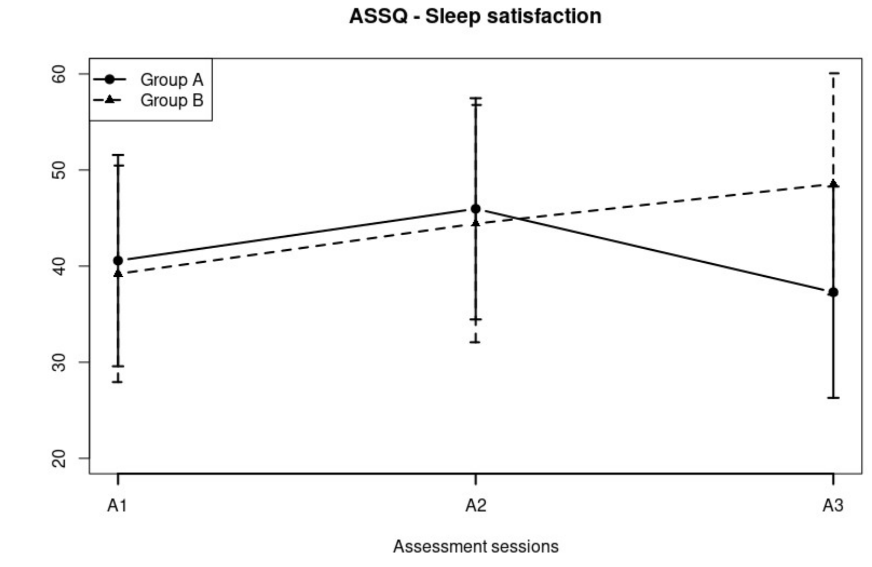

Being in shape

	Session		Group		Session * Group	
	df	*F*	*df*	*F*	*df*	*F*
Physical shape	2, 74.058	0.0633	1, 39.519	0.3661	2, 74.058	1.5271
Ability to recover	2, 73.230	0.3892	1, 39.569	1.1374	2, 73.230	1.6739
Feeling of control	2, 72.052	1.2522	1, 38.721	0.0014	2, 72.052	3.3413 *
Mental balance	2, 74.270	2.5148	1, 39.831	0.1535	2, 74.270	1.4458
Confidence	2, 71.821	0.4413	1, 39.016	0.0168	2, 71.821	1.2661
Commitment	2, 71.765	1.6611	1, 37.745	0.0002	2, 71.765	0.1929
Focus	2, 71.549	4.2797 *	1, 37.676	0.1074	2, 71.549	0.0958
Irritation	2, 73.887	0.1098	1, 39.553	1.3433	2, 73.887	0.8397
External factors affecting performance	2, 74.335	0.4917	1, 39.987	0.6506	2, 74.335	0.0991
Mental shape	2, 74.139	0.7572	1, 40.065	0.6730	2, 74.139	1.4998
Ability to suppress distraction	2, 73.898	1.2805	1, 38.826	0.2433	2, 73.898	0.4439
Flow	2, 73.506	0.6051	1, 40.094	1.1303	2, 73.506	4.1830 *
Resilience to stress	2, 72.016	1.8746	1, 38.514	0.1901	2, 72.016	0.0047
Ability to follow instructions	2, 71.865	3.8924 *	1, 38.625	3.9460	2, 71.865	0.9186
Ability to collaborate with others	2, 70.367	0.5452	1, 36.667	0.3671	2, 70.367	0.1238

$*p < 0.05$ $**p < 0.01$ $***p < 0.001$

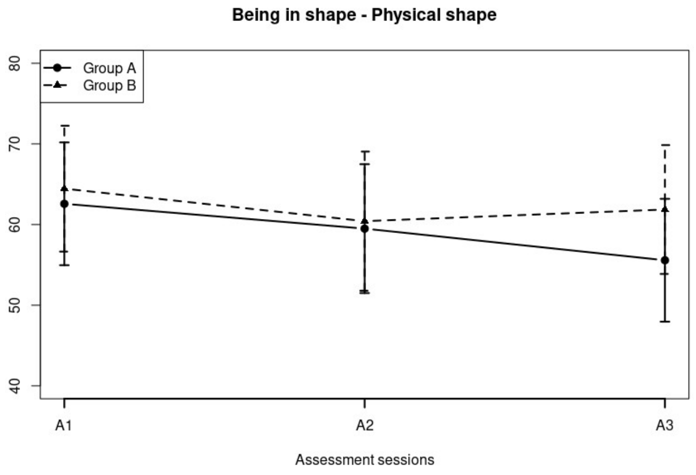

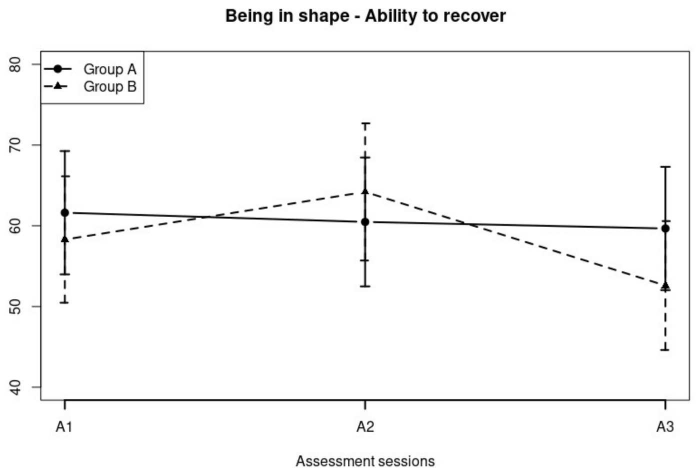

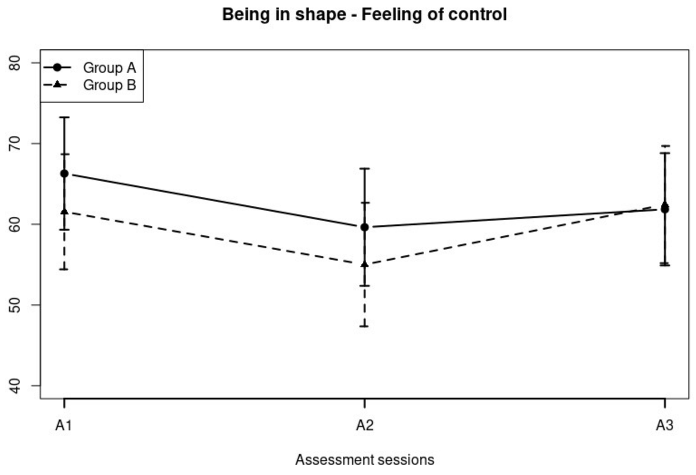

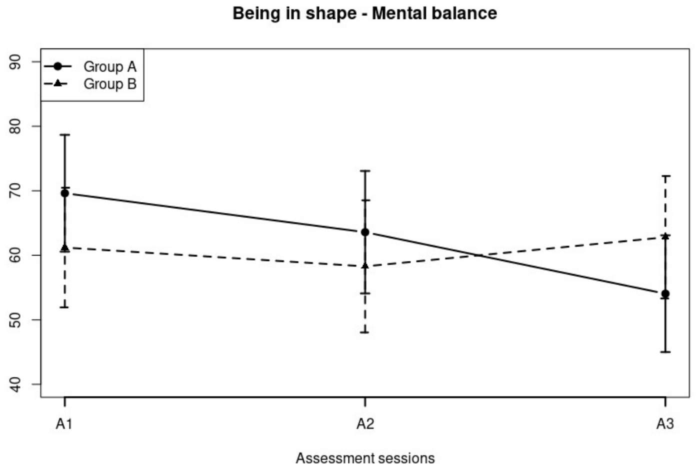

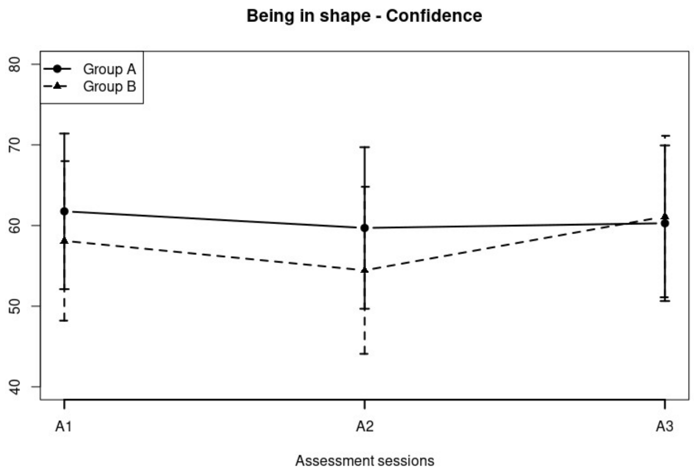

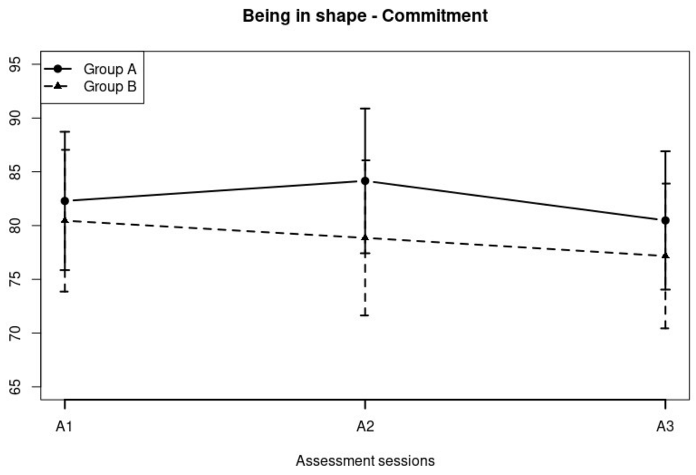

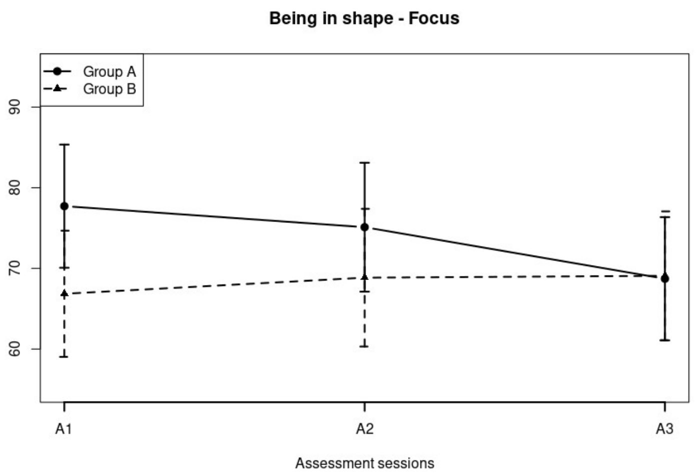
Being in shape - Focus
Group A
Group B
60
70
80
90
A1
A2
A3
Assessment sessions

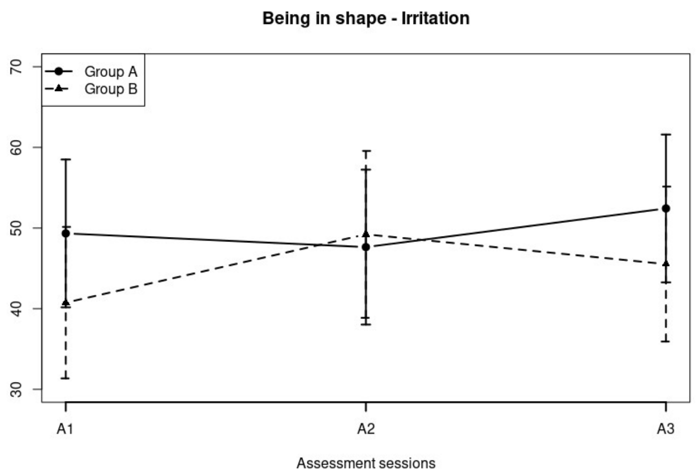
Being in shape - Irritation
Group A
Group B
30
40
50
60
70
A1
A2
A3
Assessment sessions

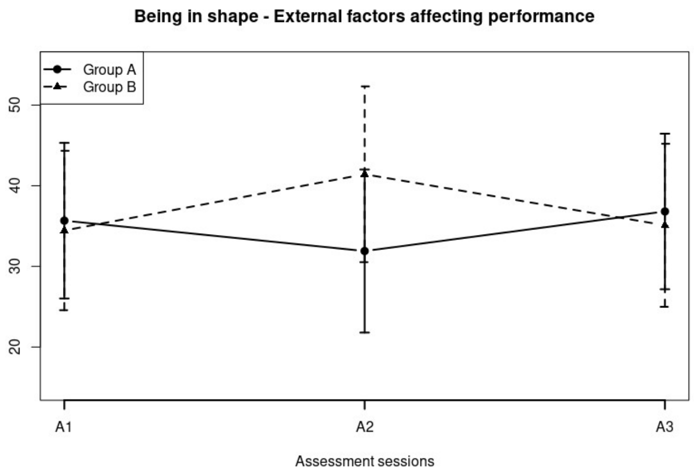
Being in shape - External factors affecting performance
Group A
Group B
20
30
40
50
A1
A2
A3
Assessment sessions

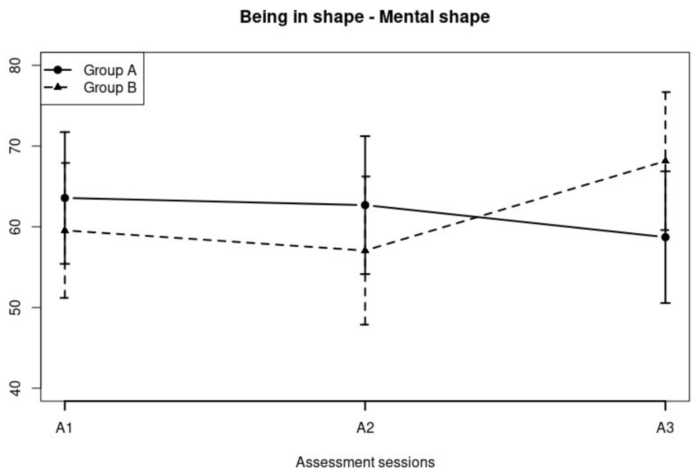
Being in shape - Mental shape
Group A
Group B
40
50
60
70
80
A1
A2
A3
Assessment sessions

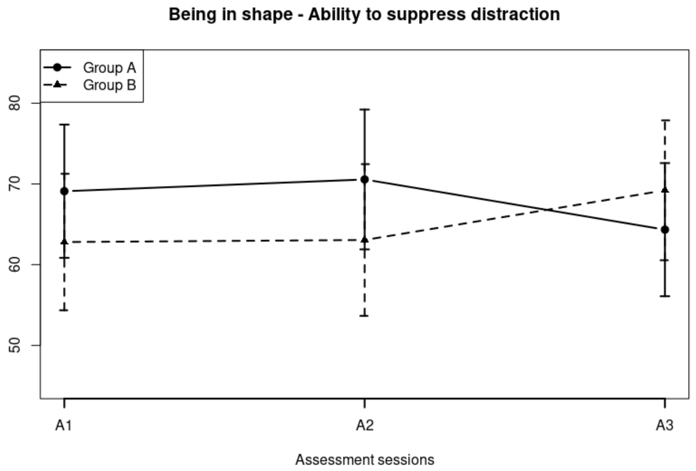
Being in shape - Ability to suppress distraction
Group A
Group B
50
60
70
80
A1
A2
A3
Assessment sessions

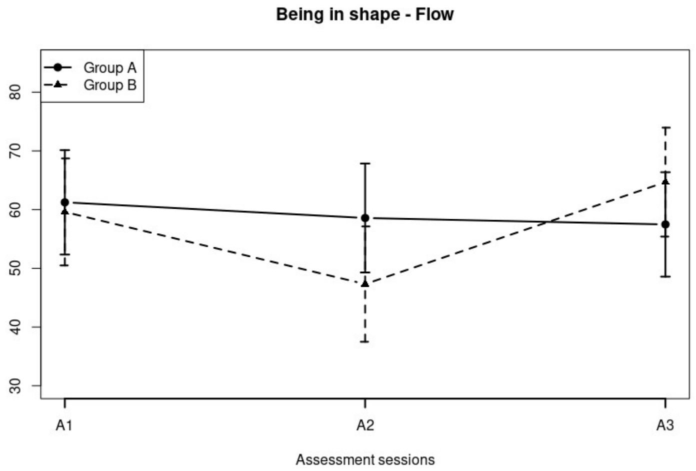
Being in shape - Flow
Group A
Group B
30
40
50
60
70
80
A1
A2
A3
Assessment sessions

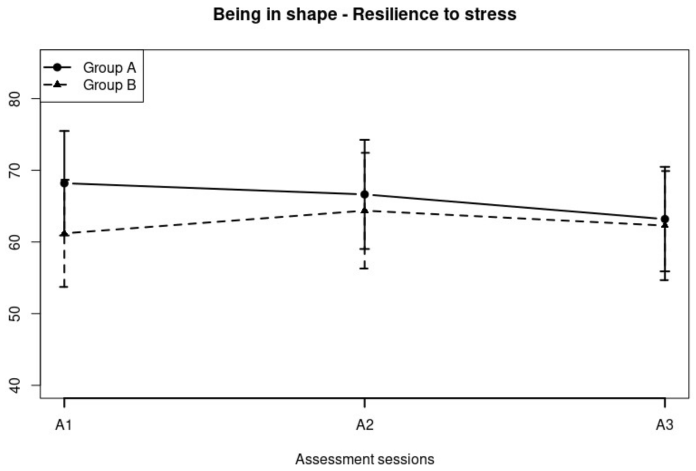
Being in shape - Resilience to stress
Group A
Group B
40
50
60
70
80
A1
A2
A3
Assessment sessions

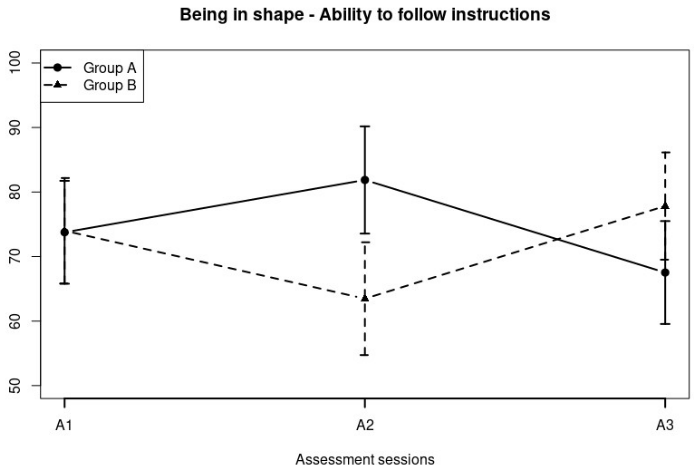
Being in shape - Ability to follow instructions
Group A
Group B
50
60
70
80
90
100
A1
A2
A3
Assessment sessions

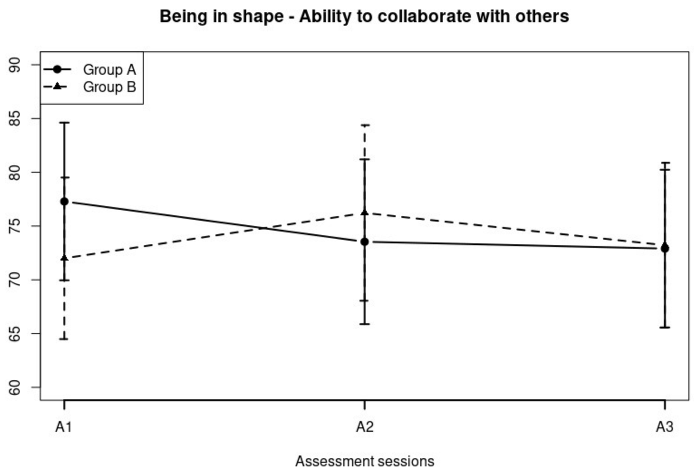
Being in shape - Ability to collaborate with others
Group A
Group B
60
65
70
75
80
85
90
A1
A2
A3
Assessment sessions

Visual-analog scale, post - pre

	Session		Group		Session * Group	
	df	*F*	*df*	*F*	*df*	*F*
Drowsiness	2, 72.695	0.8315	1, 39.969	3.7039	2, 72.695	1.6026
Relaxation	2, 68.082	0.4965	1, 37.735	0.7611	2, 68.082	0.8710
Boredom	2, 72.337	1.5537	1, 39.716	0.4127	2, 72.337	0.5316

*$p < 0.05$ **$p < 0.01$ ***$p < 0.001$

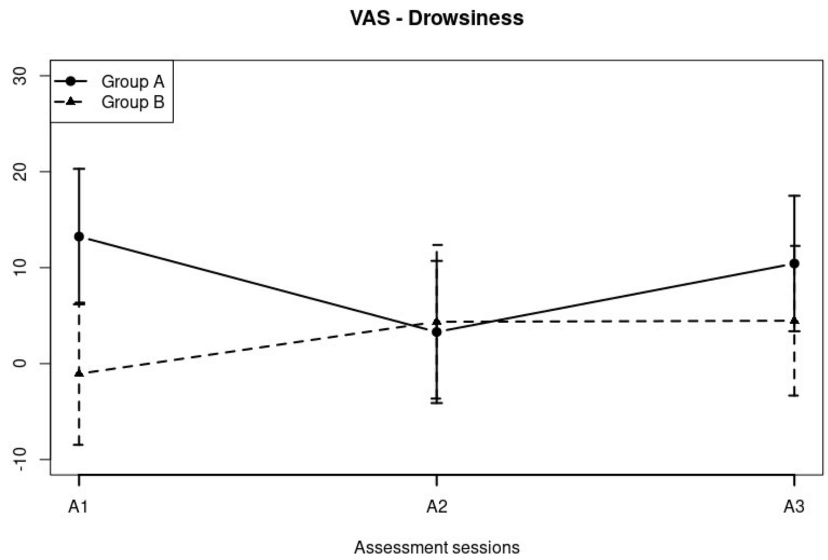

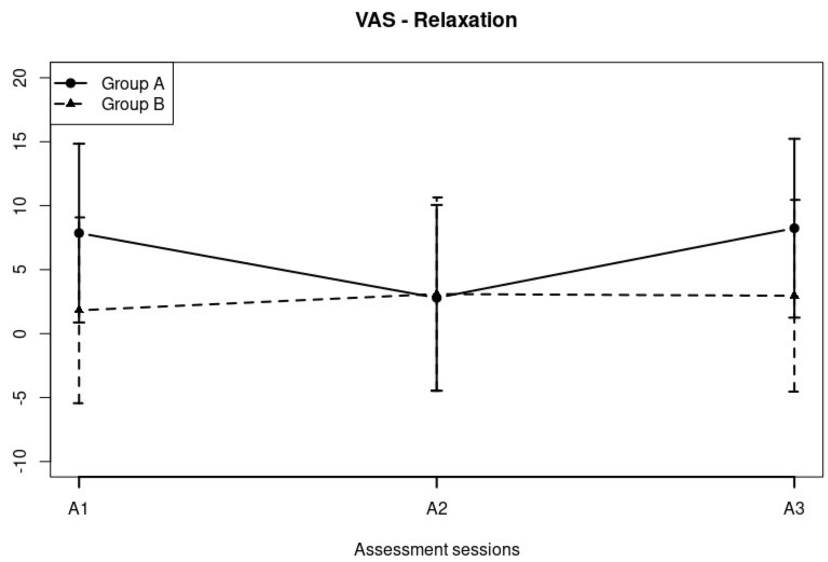

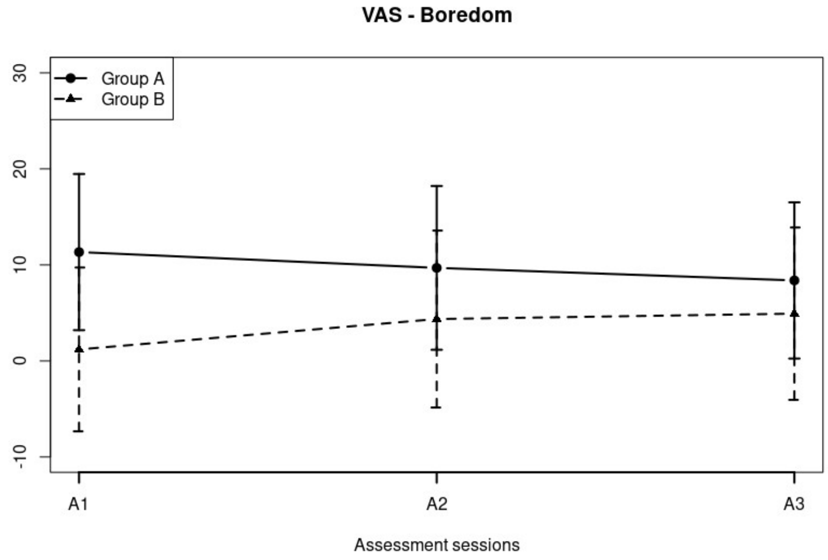

Supplement: Supplementary file 2 — Supplementary file2 (PDF 937 KB) [file 10484_2024_9654_MOESM2_ESM.pdf]
